# Uncaging Iridium(III) Complexes for Combined Photodynamic and Photoactivated Chemotherapy

**DOI:** 10.1002/anie.9954997

**Published:** 2026-08-12

**Authors:** Victoria V. L. Müller, Dominik Moreth, Irene Regeni, Lan Wang, Sylvestre Bonnet, Ulrich Schatzschneider

**Affiliations:** ^1^ Department of Chemistry Princeton University Princeton USA; ^2^ Institut für Anorganische Chemie Julius‐Maximilians‐Universität Würzburg Würzburg Germany; ^3^ Faculty of Chemistry and Biochemistry Ruhr‐Universität Bochum Bochum Germany; ^4^ Leiden Institute of Chemistry Universiteit Leiden Leiden; ^5^ Institut de science et d'ingénierie supramoléculaires (ISIS) University of Strasbourg & CNRS Strasbourg France

**Keywords:** cancer, iridium, metallodrug, photoactivated chemotherapy, photodynamic therapy

## Abstract

Inorganic photochemotherapy enables precise spatial and temporal control over anticancer drug activation, offering a promising strategy to reduce systemic toxicity. Two iridium(III) complexes of general formula [Ir(L)(ppy)(terpy)]PF_6_ with L = *N*‐acetyl‐l‐cysteinato methyl ester (cys) or *N*‐acetyl‐l‐selenocysteinato methyl ester (sec), were developed as light‐activatable prodrugs with sulfur‐ and selenium‐based “caging” groups. Both complexes are stable in the dark but undergo light induced bond cleavage upon visible light activation, following distinct photochemical pathways dictated by the coordinated chalcogen. For the cys complex, an initial oxygen‐dependent formation of a sulfinato intermediate is followed by Ir─S bond cleavage. In contrast, the selenium complex first releases dehydroalanine due to Se─C bond cleavage, forming an unprecedented selenite intermediate [Ir(SeO_3_)(ppy)(terpy)], which was characterized by single crystal x‐ray diffraction. The photoactivation leads to high singlet oxygen production quantum yields (40%–48%), consistent with a type II photodynamic therapy (PDT) mechanism. Both complexes exhibit minimal dark toxicity (EC_50 _> 150 µM) but potent photocytotoxicity in A549 cells under blue and green light photoactivation (460 and 520 nm). This new class of photocaged iridium(III) pro‐sensitizer shows promise for combined PACT/PDT applications and demonstrates how chalcogen coordination modulates the photoreactivity and therapeutic efficacy of metal complexes.

## Graphical abstract

Light‐activatable iridium(III) anticancer prodrugs demonstrate how chalcogen coordination controls photoinduced therapeutic activity. Upon visible‐light irradiation, sulfur‐ and selenium‐based caging ligands undergo distinct oxygen‐dependent photoreactions that trigger ligand release and singlet oxygen generation, enabling combined photoactivated chemotherapy (PACT) and photodynamic therapy (PDT).

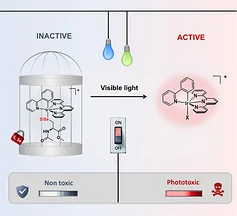

## 1 Introduction

Inorganic photochemotherapy is an emerging field for improving the selectivity of anticancer drugs and limiting side effects by local drug activation in cancer cells through light [1, 2]. Within this field, photodynamic therapy (PDT) and photoactivated chemotherapy (PACT) are the two most important treatment strategies [3, 4]. PDT agents are well‐known for catalytically generating singlet oxygen and other reactive oxygen species (ROS) upon illumination. Transition metal complexes have demonstrated significant potential in PDT, with some compounds already in clinical use [5, 6, 7]. In contrast, PACT involves light‐triggered activation of “caged” prodrugs, where a chemical bond is broken by light, releasing two fragments that are together more toxic than the prodrug [8, 9]. Either the ligand or the metal fragment (or both) can exert cytotoxic activity towards cancer cells [10, 11, 12, 13, 14, 15, 16]. The development of drugs that combine the properties of PDT and PACT within a single molecule presents an opportunity to achieve synergistic effects and enhanced therapeutic control [17, 18, 19, 20]. To date, research on such combined light‐activated PACT/PDT systems has mainly focused on Ru(II) and Pt(IV) complexes [4, 17]. For example, light activation of Pt(IV) prodrugs triggers the release of axial ligands that act as photosensitizers, while the platinum center is reduced to the cytotoxic Pt(II) stage [21, 22, 23]. In another approach, ruthenium complexes can serve as photosensitizers while undergoing photoinduced ligand exchange [19, 24]. Additionally, more complex approaches have focused on the design of binuclear compounds, where distinct parts of the molecule are responsible for the PDT and PACT properties [23, 25, 26].

While ruthenium polypyridyl complexes have dominated the development of PACT agents [12, 16, 27], analogous 5*d* ^6^ metal complexes based on Ir(III) remain less explored [28]. Iridium(III) complexes have been extensively studied as bioimaging probes and photosensitizers in PDT [29, 30, 31]. Previous work has largely focused on cyclometalated Ir(III) complexes operating via type II pathways, generating singlet oxygen (^1^O_2_) through energy transfer. However, these systems can also undergo photoinduced electron‐transfer processes to produce ROS such as superoxide and hydroxyl radicals, enabling activity under hypoxic conditions [32, 33, 34, 35]. However, their potential for photoinduced ligand exchange has received very limited attention, as Ir(III) complexes are usually characterized by high photostability [36]. Nevertheless, their photochemical properties can be fine‐tuned to enable controlled ligand release, making them promising candidates for photochemical therapeutics with combined PDT/PACT activity (Figure 1A) [37, 38].

**FIGURE 1:**
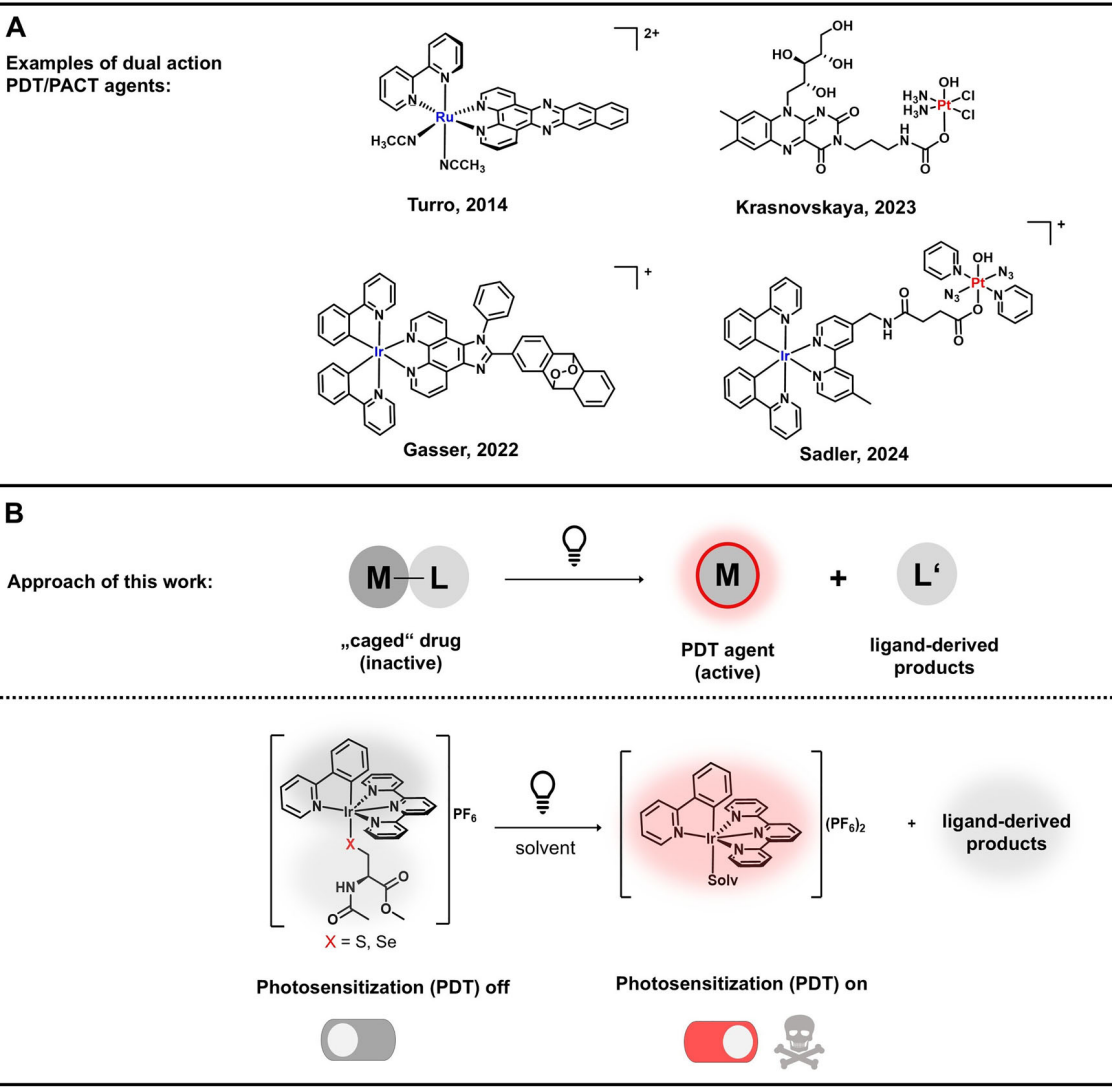
(A) Recently published examples of metal complexes as dual‐action PACT/PDT agents [22, 23, 24, 37]. (B) Strategy followed in this work for the design of Ir(III)‐based combined PACT/PDT agents.

This study presents two novel “caged” iridium(III) complexes capable of combined PACT and PDT featuring a [3+2+1] coordination sphere with N/C‐protected cysteine and selenocysteine derivatives as biogenic monodentate ligands. While biological thiols and thioethers have already been explored as metal‐caging groups [39], the selenium homologues are much less explored due to the high sensitivity of selenols towards oxidation. Our new systems provide the opportunity to study the influence of the chalcogen on photophysical and photochemical properties, with a particular focus on the mechanism of light‐triggered ligand release and the ability to generate ROS, en route to novel metal anticancer drug candidates (Figure 1B).

## 2 Results and Discussion

### 2.1 Synthesis and Characterization

Starting from [IrCl(ppy)(terpy)]PF_6_ (**1**) [36, 40], thermal exchange of the chlorido ligand with the N,C‐protected amino acids *N*‐acetyl‐L‐cysteine methyl ester (**2**) or *N*‐acetyl‐L‐selenocysteine methyl ester (**3**) led to iridium(III) complexes [Ir(cys)(ppy)(terpy)]PF_6_ (**4**) and [Ir(sec)(ppy)(terpy)]PF_6_ (**5**), where cys and sec denote the coordinated cysteinato and selenocysteinato derivates of **2** and **3**, ppy = 2‐(2‐pyridyl)phenide, and terpy = 2,2′:6′,2″‐terpyridine. In this procedure, the chlorido complex was heated under argon with an excess of the amino acid in a mixture of ethanol and water for 22 h (Figure 2A). The products were isolated as their hexafluorophosphate salts in ∼50% yield and characterized by NMR spectroscopy, mass spectrometry, and elemental analysis (see Supporting Information).

**FIGURE 2:**
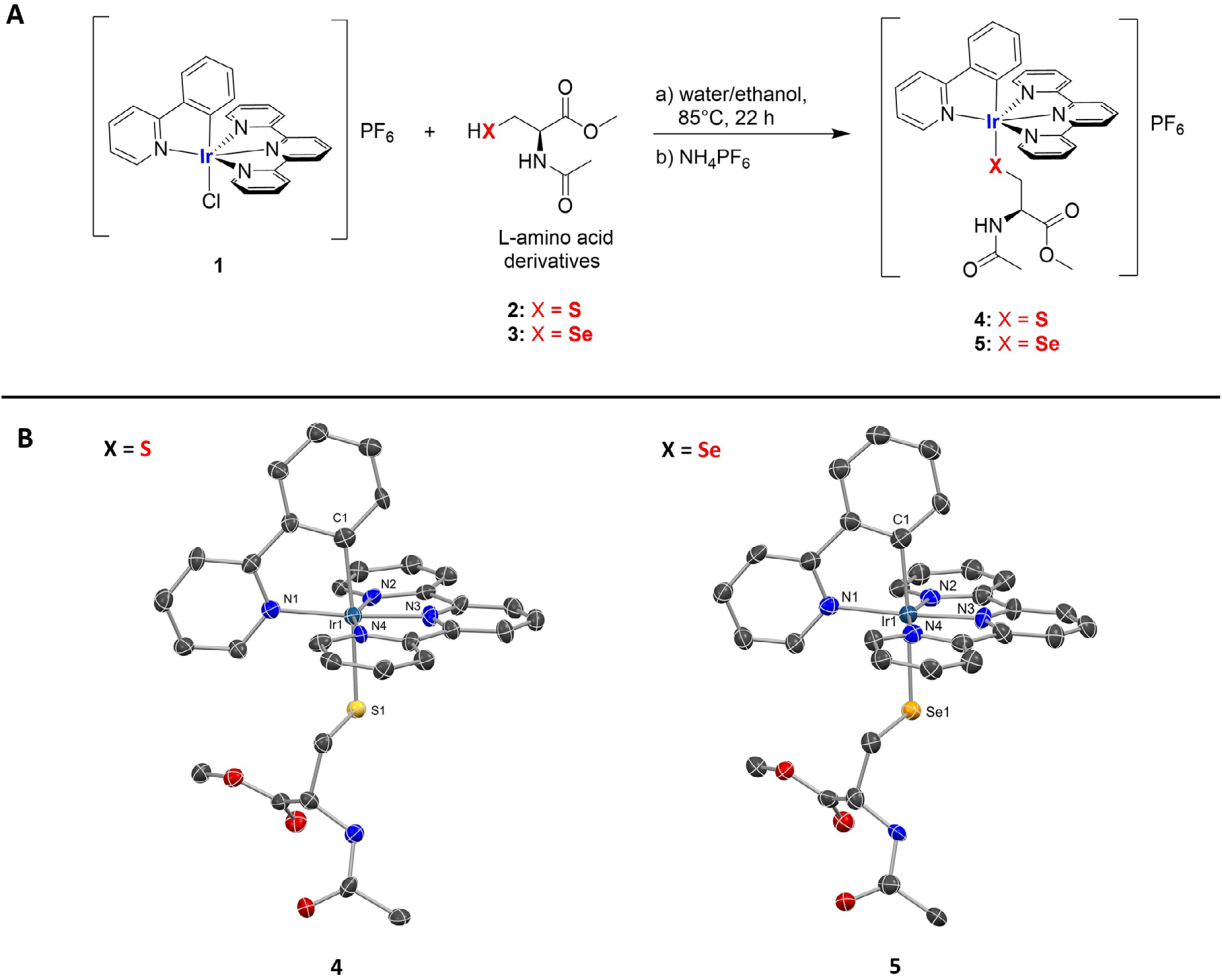
(A) One‐step synthesis of [Ir(cys)(ppy)(terpy)]PF_6_ (**4**) and [Ir(sec)(ppy)(terpy)]PF_6_ (**5**) by reaction of chlorido complex (**1**) with an excess of the corresponding amino acid derivatives **2** or **3** in a mixture of ethanol and water. (B) Molecular structures of [Ir(L)(ppy)(terpy)]PF_6_ with L = cysteinato (**4**, left) and L = selenocysteinato (**5**, right) with thermal displacement ellipsoids drawn at the 30% probability level. The hexafluorophosphate counterion, hydrogen atoms, and non‐coordinated solvent molecules are not shown for clarity.

The ^1^H NMR spectra of the two title compounds **4** and **5** show 14 aromatic signals for the protons of the two polypyridyl ligands. The signal of the ppy‐*H*_6_ proton in the pyridine ring is particularly characteristic, as it undergoes a characteristic shift upon exchange of the coordinated monodentate ligand. In chlorido complex **1**, this signal appears at 10.02 ppm, but upon cysteine coordination, it shifts to 10.21 ppm in **4**, while in the selenocysteine complex **5**, the peak appears at 10.32 ppm. The successful ligand exchange was further confirmed by the absence of the thiol (1.77 ppm) or selenol (−0.32 ppm) triplets in the aliphatic region (Figure S2 and S8). Furthermore, the ^77^Se NMR shows a shift from −66.93 ppm in free selenol **3** to −59.98 ppm in metal complex **5** (Figure S12) [41]. Single crystals suitable for X‐ray structure determination were obtained by diffusion of diethyl ether into saturated acetonitrile solutions of **4** and **5** at 4 °C (Figure 2B and Supporting Information). Both complexes crystallized in the chiral space group *P*2_1_, indicating the enantiopure nature of the compounds (also see the Flack parameter reported in the Supporting Information). The metal‐chalcogen distances in the crystal structure are distinct: 2.443(5) Å for the Ir─S bond distance versus 2.560(3) Å for the Ir─Se bond distance. For comparison, an octahedral iridium complex featuring S‐phenylated cysteine coordinated as an N,S‐chelating ligand was previously reported with an Ir─S bond length of 2.4084(17) Å, which is comparable to the monodentate S‐coordination observed in complex **4** [42].

### 2.2 Photophysical Characterization and Photoactivation Studies by UV/Vis and Fluorescence Spectroscopy

Effective photoactivatable drug candidates need to be stable in the dark, which can be checked by UV/Vis spectroscopy. The absorption spectra of **4** and **5** in acetonitrile were found to be almost identical, with intense absorption bands in the range of 200–350 nm and weaker absorptions extending up to 600 nm (Figure 3C). Sulfur versus selenium coordination results in a weak bathochromic shift of the low‐energy absorption band from 380 to 390 nm, with almost identical molar extinction coefficients (*ε*_380_ = 0.41 × 10^4^ M^−1^ cm^−1^ for **4** vs. *ε*_390_ = 0.39 × 10^4^ M^−1^ cm^−1^ in **5**). Both complexes exhibit good dark stability under biologically relevant conditions, showing no detectable changes in their UV/Vis spectra after 48 h in acetonitrile, water, or OptiMEM cell‐culture medium, and no ligand substitution or decomposition was observed in the presence of excess glutathione (Figures S23 and S24). Upon illumination with UV (385 nm), blue (450 nm), and green (515 nm) light, both iridium complexes showed changes in the absorption spectra, whereas no photoreactivity was observed under red light exposure (625 nm) within the studied time frame (Figures 3A and S25–S38). While the time evolution of the UV/Vis spectra indicates isosbestic points, kinetic analysis clearly shows that the photochemistry does not follow the first‐order rate law expected for simple photosubstitution, suggesting the involvement of intermediate species. Since UV/Vis spectroscopy did not allow the identification of the photoactivated products, ESI mass spectra were recorded following illumination of the complexes. In acetonitrile, photoactivation led to the formation of the corresponding dicationic acetonitrile complex [M–X–2PF_6_+CH_3_CN]^2+^ (with X = cys or sec) at *m/z* = 310.7, along with a signal for [IrCl(ppy)(terpy)]^+^, possibly formed in the mass spectrometer (Figures S25–S38). On the other hand, when illuminated in water, the dicationic aqua complex [M–X–2PF_6_+H_2_O]^2+^ at *m/z* = 299.1 was identified as the major photoproduct. Photoactivation can also be followed by emission spectroscopy. While both complexes exhibited only weak emission in acetonitrile in the dark (*λ*_em, max_ = 544 nm), illumination resulted in a seven‐fold enhancement of the emission intensity without changing the emission maximum (Figures 3B and S39–S41). Consistent with this observation, the emission quantum yield increased from 4.8% (**4**) and 5.4% (**5**) in the caged state to approximately 35% after photoactivation. Similar behavior was observed under physiological conditions in Opti‐MEM cell culture medium, where the emission intensity increased progressively with the applied light dose (*λ*_illum_ = 460 nm, 19.2 mW/cm^2^) (Figure S42). Overall, the turn‐on emission following light activation suggests that fluorescence microscopy could be used to detect the activated metal fragment in cells (see the following section). To investigate the potential of both iridium complexes as photosensitizers for type II PDT, singlet oxygen quantum yields (*Φ*_Δ_(^1^O_2_)) were determined from the emission of ^1^O_2_ at 1270 nm in acetonitrile under blue light illumination (450 nm) using perinaphthenone as a reference (*Φ*_ref_Δ = 0.98) [43, 44]. Interestingly, both complexes generated almost no singlet oxygen before light activation (Figure 3E and Table S5). The low but non zero value of *Φ*_Δ_(^1^O_2_) = 0.05 for the cysteinato complex is probably due to the experimental setup, since the short laser shot used to perform the measurement already induced some ligand cleavage and release of photodynamically active complex **1** (Figure S45). After complete photoactivation, both complexes exhibit a pronounced singlet oxygen emission with quantum yields of 0.40 (**4**) and 0.48 (**5**), compared to 0.52 for **1** (Table S5). Therefore, the caged complexes are initially inactive, but generate PDT‐active species comparable to reference complex **1** after photolysis. To further assess whether the iridium complexes exhibit type I PDT activity, superoxide radical (O_2_^·−^) generation was determined using dihydroethidium (DHE) as a chemoselective probe. Both complexes generated superoxide upon illumination, with cysteinato complex **4** showing efficient activity comparable to reference complex **1** under blue light illumination. In contrast, the selenium analogue **5** exhibited lower superoxide production, which is the result of a slower photodissociation of the Ir─Se bond relative to the Ir─S bond in **4** to the uncaged active species. Green light illumination was generally less effective for all three complexes.

**FIGURE 3:**
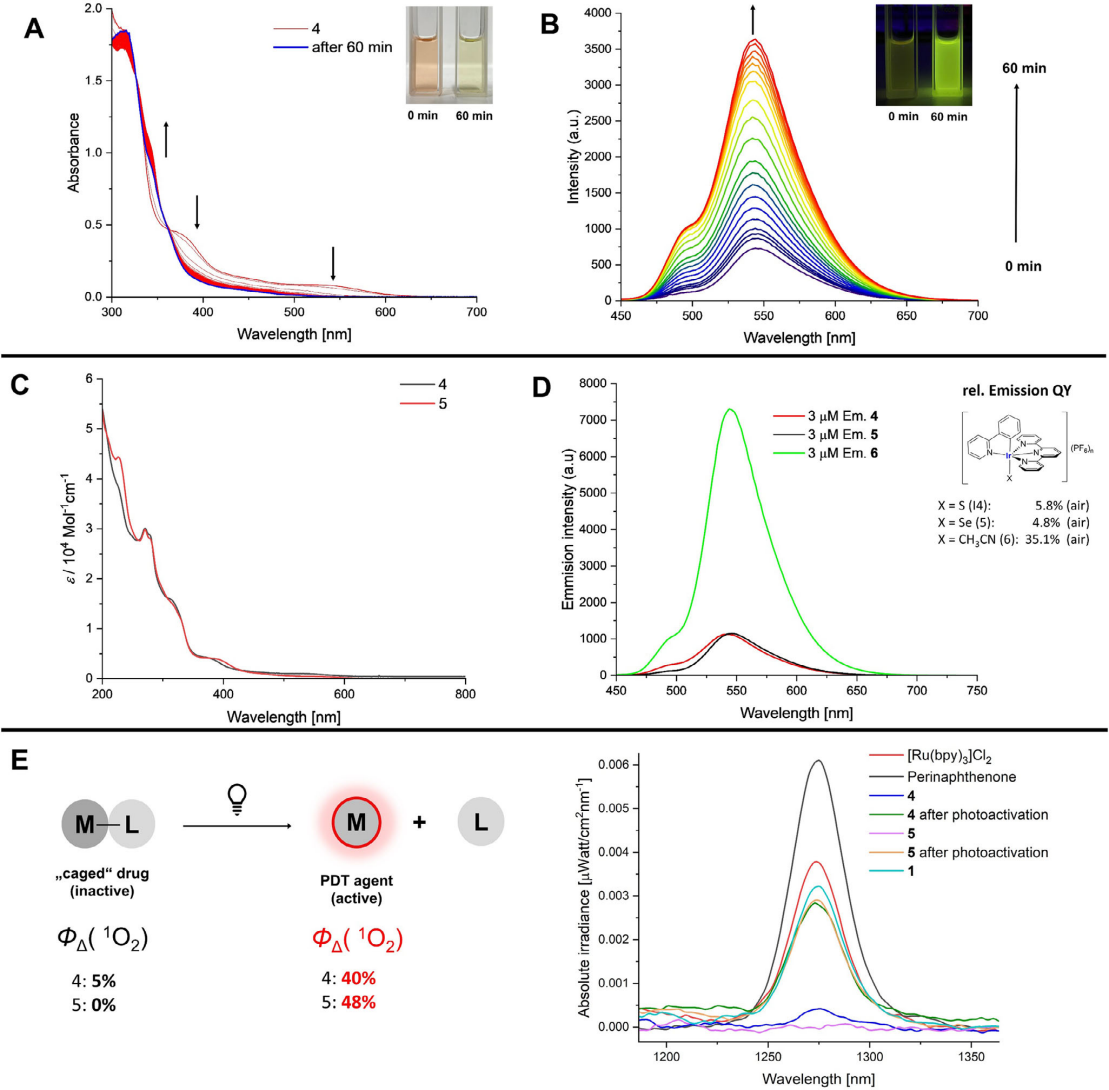
(A) Changes in the UV/Vis spectra of **4** in acetonitrile under 450 nm illumination. The inset shows a photograph of a 150 µM solution of the complex before (left) and after illumination (right). (B) Changes in the emission spectra of **4** in acetonitrile under 385 nm illumination. The inset shows a 150 µM solution of the complex before (left) and after illumination (right). (C) UV/Vis spectra of **4** (black) and **5** (red) in acetonitrile at room temperature. (D) Comparison of the emission spectra of **4**, **5**, and the photoactivation product [Ir(CH_3_CN)(ppy)(terpy)](PF_6_)_2_ (**6**) in acetonitrile (3 µM). (E) Switching on the singlet oxygen production by light. The ^1^O_2_ generation quantum yield determination based on the NIR emission at 1270 nm are indicated before and after light activation under 450 nm light. The spectra on the right shows NIR emission spectra for reference perinaphthenone (black), [Ru(bpy)_3_]Cl_2_ (red), and for complexes (**1**, cyan), non‐activated **4** (blue), activated **4** (green), non‐activated **5** (pink), and activated **5** (orange), all measured in air‐saturated acetonitrile.

### 2.3 Photoactivation Studies by ^1^H NMR Spectroscopy and ESI Mass Spectrometry

To identify the photoactivation products and elucidate the underlying reaction mechanism, the photoreactions of complexes **4** and **5** were investigated by ^1^H NMR spectroscopy and ESI mass spectrometry in acetonitrile under green light illumination (520 nm). The reaction progress was tracked by following the diagnostic ppy‐*H*_6_ resonance at 10.21 ppm (**4**) or 10.32 ppm (**5**), which is highly sensitive to changes in the monodentate ligand. Upon illumination of **4**, the signal of the starting material completely disappeared within 6 min and was replaced by two new signals for ppy‐*H*_6_ at 10.19 and 9.35 ppm. Comparison with an independently synthesized sample identified the latter as the solvent species [Ir(CH_3_CN)(ppy)(terpy)](PF_6_)_2_ (**6**), which became the exclusive species present after 30 min of illumination (Figure 4). The signal at 10.19 ppm is due to an unknown intermediate **7**, which was further characterized by additional 2D ^1^H NMR experiments.

**FIGURE 4:**
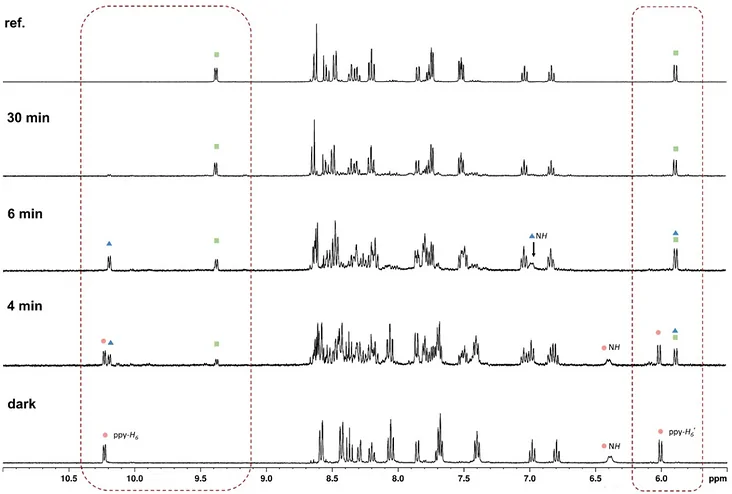
^1^H NMR spectra of **4** (2.5 mM) in CD_3_CN before and after illumination with a green LED light (520–525 nm, 0–30 min). The spectrum of the acetonitrile complex **6** is as shown at the top as a reference. The observed species are assigned to **4** (red dot), an intermediate iridium species (blue triangle), and **6** as the final product (green squares).

In complex **4**, the proximity between the two chemically non‐equivalent methylene protons of the cysteinato ligand and the ppy‐*H*_6_ of the pyridine ring in the ppy ligand results in two cross peaks in the ^1^H,^1^H NOESY spectrum (Figure 5, left). The ^1^H,^1^H NOESY spectrum of the illuminated sample recorded in CD_3_CN also shows a cross peak between ppy‐*H*_6_ and the aliphatic methylene resonances of the monodentate ligand at 3.43 ppm (Figure 5, right; Figures S49 and S50). This NOE correlation clearly demonstrates that the cysteinato ligand remains coordinated to the iridium center in intermediate **7**. The high‐resolution ESI mass spectrum of a sample illuminated with green light for 6 min showed a monocationic species at *m/z* = 788.1525, which is assigned as the sulfinato complex [Ir(cys‐O_2_)(ppy)(terpy)]^+^ based on the excellent match with a theoretical calculated mass of 788.1513 Da (Figures S54 and S55). Consistent with the ^1^H NMR spectrum, an additional peak for the dicationic acetonitrile complex **6** was also observed at *m/z* = 310.5745 (calc *m*/*z* = 310.5747, Figures S54 and S56). These observations indicated that **4** undergoes an initial oxidation to a coordinated sulfinato species, before ligand substitution affords the formation of the solvent‐coordinated species **6** (Figure 6A, top). The absence of photoreactivity upon illumination of **4** under argon clearly demonstrates that dioxygen is necessary for ligand cleavage (Figures S52 and S53). When the illumination was carried out in air in the presence of excess tetrabutylammonium chloride, the chlorido starting material was obtained, suggesting that it is also the main photoproduct in vitro (Figure S51). In contrast to **4**, complete photoactivation of the selenium analogue **5** required at least 60 min of illumination under identical conditions (Figure S58). In the final ^1^H NMR spectrum, the characteristic resonances of the acetonitrile complex **6** were identified, but some remaining aromatic signals of other unidentified species were also present. In addition, two singlets at 6.39 and 5.74 ppm, each with an integral of 1H, and two singlets at 3.79 and 2.04 ppm, each with an integral of 3H, were indicative for the formation of dehydroalanine as an elimination product (Figure 6A, bottom; Figure S59) [45]. In contrast, an ESI mass spectrum recorded after 20 min of illumination of **5** showed a complex mixture of various charged species with only limited peak assignment possible (Figure S62). Notably, no signal corresponding to an oxidized selenocysteine intermediate analogous to the sulfinato complex **7** could be experimentally detected. This demonstrates that distinctly different reaction pathways are operative for the S versus Se iridium complexes. In accordance with the ^1^H NMR spectrum of illuminated **5** in acetonitrile, ESI mass analysis identified signals corresponding to the starting material, the dicationic acetonitrile species **6**, and the chlorido species **1**. Finally, a signal at *m/z* = 709.0315 matched the simulated isotope pattern and calculated mass of *m/z* = 709.0330 of [Ir(HSeO_3_)(ppy)(terpy)]^+^, corresponding to the formation of a hydroselenite ion (HSeO_3_^−^) that remains bound to iridium (Figure S63). A crystal grown from an illuminated NMR sample was used to structurally characterize this intermediate by single crystal X‐ray diffraction (Figure 6B). The absence of a counterion and the pseudo‐tetrahedral environment of the selenium atom are consistent with a Se oxidation state of +IV and the assignment of the monodentate ligand as selenite (SeO_3_^2−^). The identification of a coordinated selenite in the intermediate [Ir(SeO_3_)(ppy)(terpy)] (**9**) further supports the release of dehydroalanine by photoactivation. Metal complexes with coordinated oxidized selenium species are extremely rare [46], and this is the first structurally characterized molecular species reported to date. Since the compound features an *O*‐coordinated selenite, a change in the coordination mode from an initially Se‐bound species must occur at some stage during the reaction, suggesting an essential photooxidation step, like for the sulfur analogue (Figure 6A, bottom).

**FIGURE 5:**
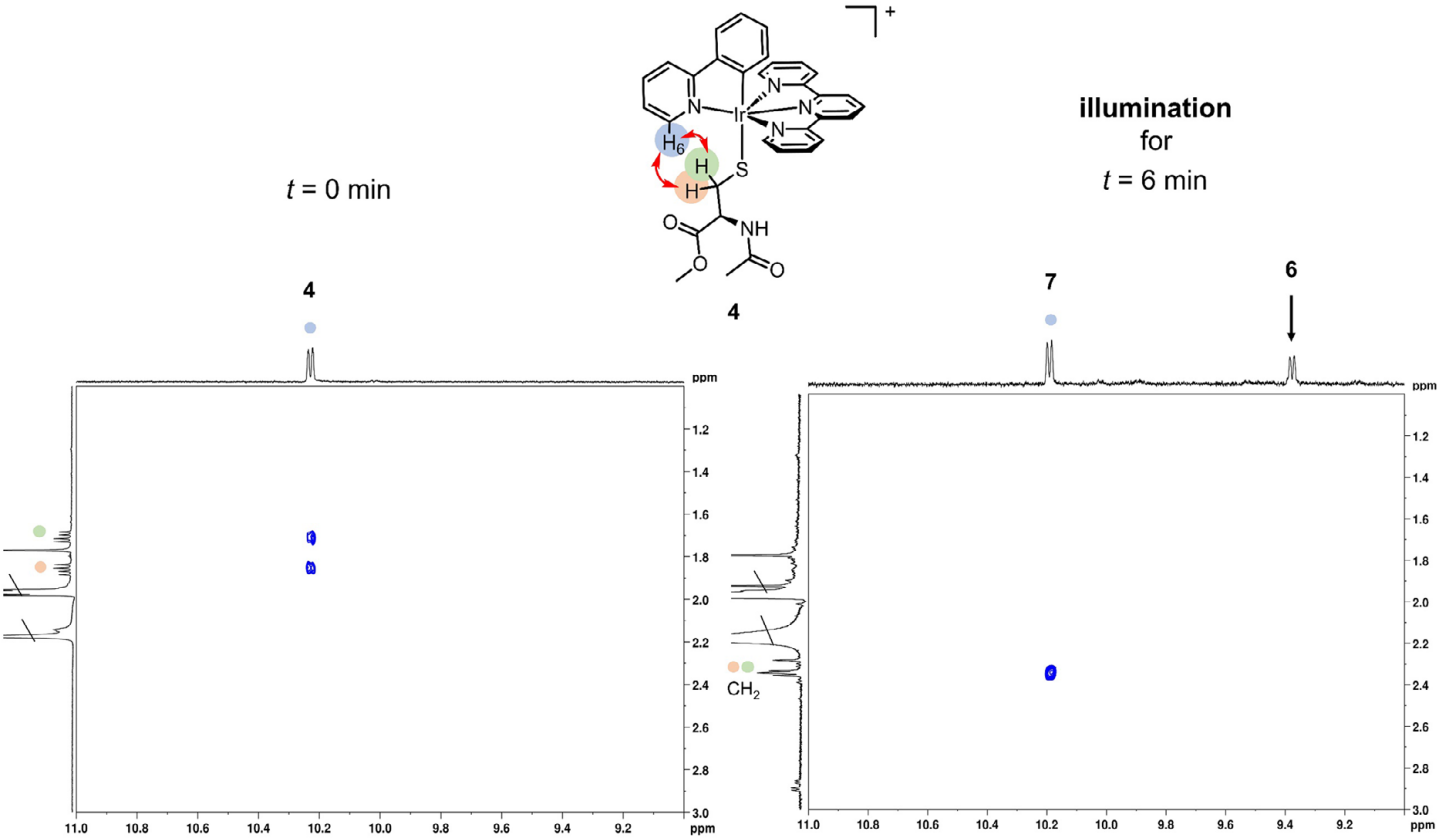
Enlarged view of the ^1^H,^1^H NOESY spectrum of **4** before (left) and after 6 min of illumination with green light (520–525 nm, right). The ppy‐*H*_6_ peak (blue dot) shows cross peaks with the aliphatic methylene group (green and orange dot) of the monodentate ligand in both 2D spectra, showing that the ligand remains attached in intermediate **7**.

**FIGURE 6:**
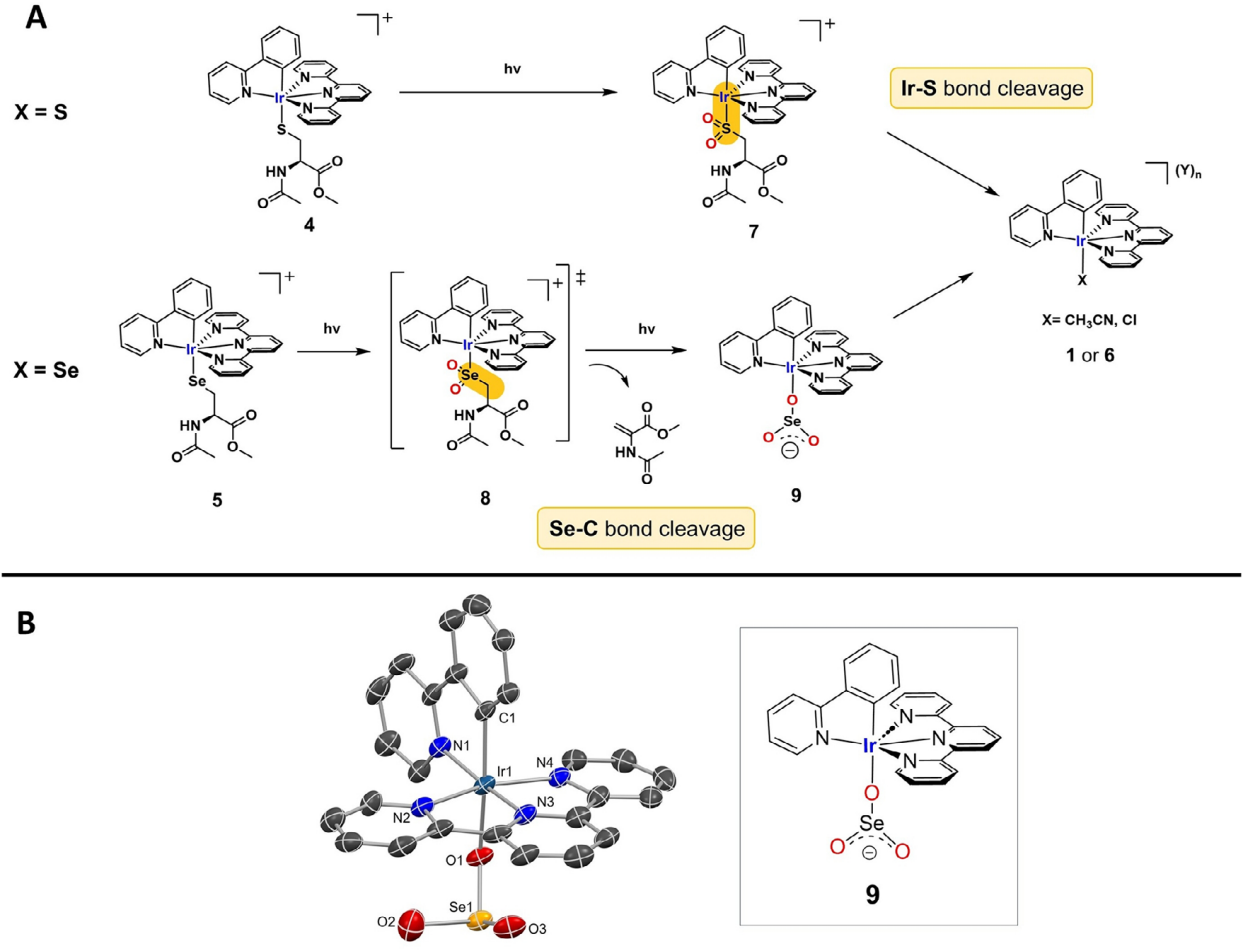
(A) Proposed mechanism for the photoactivation of the title complexes **4** and **5**. In the cysteinato complex, the ligand first undergoes an oxygen‐dependent oxidation to sulfinato intermediate **7**, which is subsequently replaced by solvent or chlorido ligands from the reaction mixture (Ir─S bond cleavage). In contrast, the selenocysteine analogue forms intermediate **8**, from which dehydroalanine is eliminated via Se─C bond cleavage, accompanied by the formation of the selenite‐coordinated intermediate **9**, which is subsequently replaced by solvent or chlorido ligands. (B) Molecular structure of [Ir(SeO_3_)(ppy)(terpy)] (**9**) with thermal displacement ellipsoids drawn at the 50% probability level and the hydrogen atoms not shown for clarity.

When the photoactivation experiments were repeated for **5** under argon, no reaction was observed even after extended illumination (Figure S61), indicating that photoactivation is oxygen‐dependent, similar to **4**. In the presence of excess chloride, the reaction proceeded more rapidly and afforded chlorido complex **1** as the final product (Figure S60). Thus, selenite species **9** appears to be an intermediate formed upon light activation of **5** under aerobic conditions, whereas acetonitrile complex **6** and chlorido complex **1** represent the final photoproducts, depending on the chloride concentration.

### 2.4 Quantum Chemical Calculations

To get insight into the photoinduced ligand dissociation mechanism, DFT and TD‐DFT calculations were performed. Hirshfeld charge analysis and Mayer bond order calculations were performed for the optimized ground‐state structures to evaluate the polarity and relative strength of the Ir–ligand bonds (Table 1). The cysteine and selenocysteine complexes **4** and **5** show positive charge separation values, Δ*q*(Ir–L), of +0.455 and +0.426, respectively, consistent with polarized Ir─S and Ir─Se bonds. Mayer bond order analysis further indicates a substantially weaker Ir–Se interaction compared to the Ir–S analogue, with a Mayer bond order of 0.532 for **5** compared to 0.804 for **4**. Upon oxidation of the chalcogen donor, the bond polarity changes markedly. In **7** and **8,** the sulfur and selenium donor atoms become positively charged, leading to reversed Δ*q* values of −0.201 and −0.247, respectively. This reflects the strongly reduced donor character of the oxidized chalcogen ligands. Nevertheless, the Ir–Se interaction remains weaker than the Ir–S interaction, as shown by the lower Mayer bond order of **8** compared to **7**. This reflects the reduced donor character of the oxidized chalcogen ligands and identifies these species as electronically weakened intermediates that are more susceptible to ligand substitution. The SeO_3_ complex **9** represents a distinct bonding situation, as the ligand coordinates through oxygen rather than selenium. Hirshfeld analysis gives Δ*q*(Ir–O) = +0.595, indicating a strongly polarized Ir─O bond, while the relatively low Mayer bond order of 0.494 suggests limited covalent stabilization and thus a possible slower pathway for chloride exchange.

**TABLE 1:** Experimental and calculated Ir─L bond distances, Hirshfeld charge differences Δ*q*(Ir─L), and Mayer bond orders for complexes **1**, **4**, **5**, and **7–9**.

Compound	M‐L distance exp. (Å)	M‐L distance calc. (Å)	Hirshfeld charge difference Δ*q*(M–L)	Mayer bond order
**1**	2.427	2.513	+0.628	0.797
**4**	2.443	2.481	+0.455	0.804
**5**	2.560	2.613	+0.426	0.532
**7**	2.134	2.128	−0.201	0.678
**8**	—	2.580	−0.247	0.491
**9**	2.142	2.143	+0.595	0.494

TD‐DFT calculations provide further insight into the different photochemical behavior of the reduced and oxidized complexes (Figure 7). For the cysteine and selenocysteine complexes **4** and **5**, the lowest excited states are dominated by HOMO→LUMO excitations. The HOMO contains substantial sulfur or selenium donor contribution, whereas the LUMO is mainly localized on the π* system of the terpyridine ligand. The corresponding electron‐density difference maps therefore show pronounced depletion of electron density at the coordinated chalcogen donor atom and accumulation on the aromatic ligand framework. This indicates a mixed chalcogen/metal‐to‐ligand charge‐transfer character and generates partial sulfur‐ or selenium‐centered radical character in the excited state. This excited‐state hole density at the coordinated thiolate or selenolate ligand facilitates the reaction with molecular oxygen and allows rationalization of the formation of oxidized species **7** and **8**. The low‐lying excited states of chalcogen complexes **4** and **5** are consistent with their pronounced chalcogen‐centered photoreactivity. In contrast, the oxidized species **7** and **8** display excited‐state electron‐density redistribution patterns that are more similar to the chloride complex **1**, indicating a reduced contribution of the chalcogen donor to the frontier orbitals. In **7** and **8**, the HOMO is mainly localized on the Ir/ppy framework with only minor contribution from the oxidized chalcogen ligand, while the LUMO remains terpyridine π*‐centered. Therefore, the excited states of **7** and **8** are best described as predominantly Ir/ppy‐to‐terpyridine charge‐transfer states with mixed MLCT/LLCT character and a strongly reduced sulfur‐ or selenium‐centered hole density. This suppresses the chalcogen‐centered excited‐state quenching pathway observed for the reduced complexes **4** and **5**. Consequently, radiative decay from the Ir(ppy)(terpy)‐based triplet state becomes competitive, assisted by strong spin–orbit coupling at the Ir(III) center, resulting in a luminescence turn‐on for the oxidized species **7** and **8**.

**FIGURE 7:**
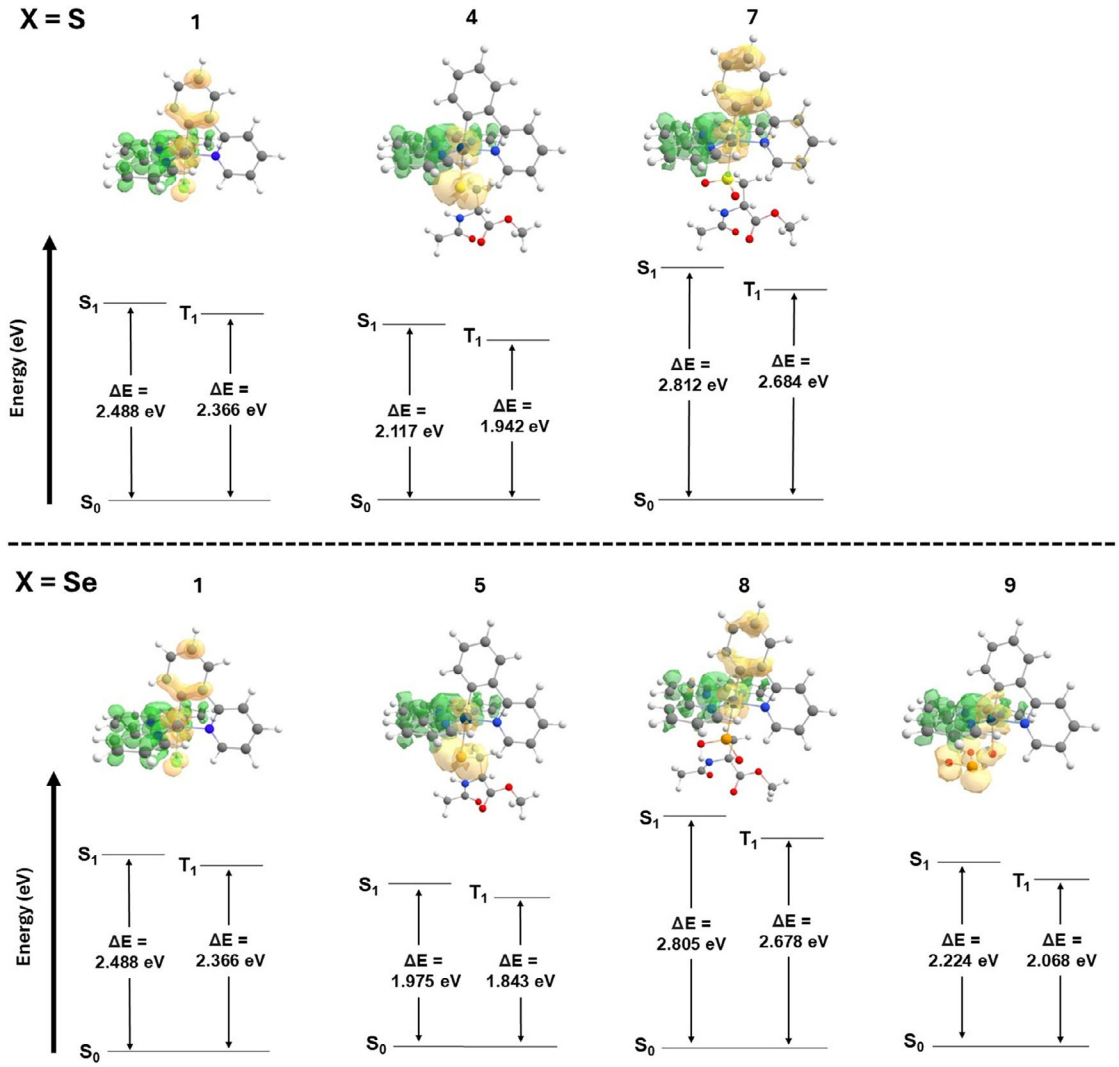
TD‐DFT electron‐density difference maps and S_1_/T_1_ excitation energies for complexes **1**, **4**, **5**, and **7–9**, including the respective calculated transition energies.

The ligand exchange can be rationalized by the combined effect of a weakened ground‐state bonding after oxidation and additional excited‐state labialization. In **7** and **8**, oxidation reduces the donor strength of the monodentate ligand and reverses the Ir─L bond polarity. Upon excitation, MLCT/LLCT redistribution from the Ir/ppy region toward terpyridine further perturbs the Ir─L bond, facilitating substitution. For sulfur, this enables efficient chloride exchange from **7** to form **1**. In contrast, **8** is more prone to further oxidative transformation, because the Ir─Se bond is weaker than the Ir─S bond in **7**, and the Se─C bond is longer than the corresponding S─C bond (1.982 Å vs. 1.826 Å). Thus, excitation can destabilize the already labile Ir─Se/Se─C framework, promoting overoxidation and Se─C cleavage to the O‐bound SeO_3_ complex **9**, whereas the stronger Ir–S and S─C bonds in **7** disfavor analogous SO_3_ formation.

### 2.5 Photocytotoxicity Studies

The initial photochemical cleavage of the Ir─S and Ir─Se bonds in complexes **4** and **5**, respectively, is typical of PACT. On the other hand, the ability of the chlorido species **1** or solvato photoproducts similar to **6** to generate ROS upon further light exposure is typical for PDT. Both pathways are combined in a unique fashion in the title compounds, calling for the investigation of the photocytotoxicity of iridium complexes **4** and **5** in cancer cells. Complex **1** was included as a stable reference for the uncaged state, based on the anticipated rapid conversion of photo‐generated solvato intermediates into the corresponding species **1** in the chloride‐rich cell culture medium. Considering the absorption spectrum of both complexes (Figure 3C), the focus was first on blue light activation. The cytotoxicity and phototoxicity of the three compounds against the human lung cancer cell line A549 was evaluated under normoxic (21% O_2_) and hypoxic (1% O_2_) conditions. Following a published in vitro protocol for photopharmaceutical testing [47], cells were treated with the compounds 24 h after seeding, incubated in the dark for additional 24 h, and then illuminated at a fixed wavelength (450 nm) and intensity (19.2 mW/cm^2^). In one set of experiments, the initial drug‐containing medium was replaced with fresh, drug‐free medium before photoactivation, while in a second protocol, the medium was not exchanged before light activation. While the former tends to minimize cytotoxicity, the latter maximizes them, while it remains to be discussed with one or the other best model what happens in a real tumor in vivo. Under both conditions, one plate was exposed to light, while another one was kept in the dark as a control. Relative cell viability was determined 48 h after light activation by MTT assay, and half‐maximal effective concentrations for cell growth inhibition (EC_50_, in µM) were calculated from the dose–response curves of treated *vs*. non‐treated wells (Tables 2 and S13; Figures 8, S85 and S86). The photoindex (PI) was calculated as the quotient of the EC_50_ values determined in the dark and under light‐exposed conditions. Both compounds **4** and **5** exhibit negligible toxicity in the dark, with EC_50_ values exceeding 150 µM. To evaluate the impact of light dosage on the photocytotoxicity of **4** and **5**, two different doses of blue light (460 nm) were tested, that is, 8 J/cm^2^ (19.2 mW/cm^2^, 7 min) and 16 J/cm^2^ (19.2 mW/cm^2^, 14 min). For both complexes, the higher light dose resulted in increased photocytotoxicity characterized by larger PI values, suggesting the involvement of photocatalytic processes typical of PDT, or incomplete stochiometric activation at the low light dose, which is typical of PACT. Under all conditions investigated, the cysteinato complex **4** was more phototoxic than the selenocysteine analogue **5** by a factor of two to five, when comparing the PI. In particular, **4** showed very promising photocytotoxicity at 16 J/cm^2^ with an EC_50, light_ value of 4.9 µM and a PI of larger than 30.

**TABLE 2:** EC_50_ values for A459 lung cancer cells exposed to **1**, **4**, or **5** in the dark and upon blue or green light illumination under normoxia (21% O_2_) determined with the MTT assay without medium exchange.

Compound	Dark		Blue (460 nm)						Green (520 nm)		
			8 J/cm^2^			16 J/cm^2^			16 J/cm^2^		
	EC_50_ (µM)	CI^b^ 95% (µM)	EC_50_ (µM)	CI^b^ 95% (µM)	PI^a^	EC_50_ (µM)	CI^b^ 95% (µM)	PI^a^	EC_50_ (µM)	CI^b^ 95% (µM)	PI^a^
**1**	36.7	−6.2 +7.8	—	—	—	3.8	−1.2 +1.5	9.6	—	—	—
**4**	> 150	—	11.5	−2.3 +2.5	> 13.1	4.9	−2.1+2.2	> 30.5	24.5	−4.0 +4.4	> 6.1
**5**	> 150	—	39.4	−7.1 +9.3	> 3.9	27.1	−5.3 +7.0	> 5.6	48.5	−8.4 +9.4	> 3.2

^a^ Photocytotoxicity index (PI) = EC_50, dark_/EC_50, light_.

^b^ CI = Confidence interval.

**FIGURE 8:**
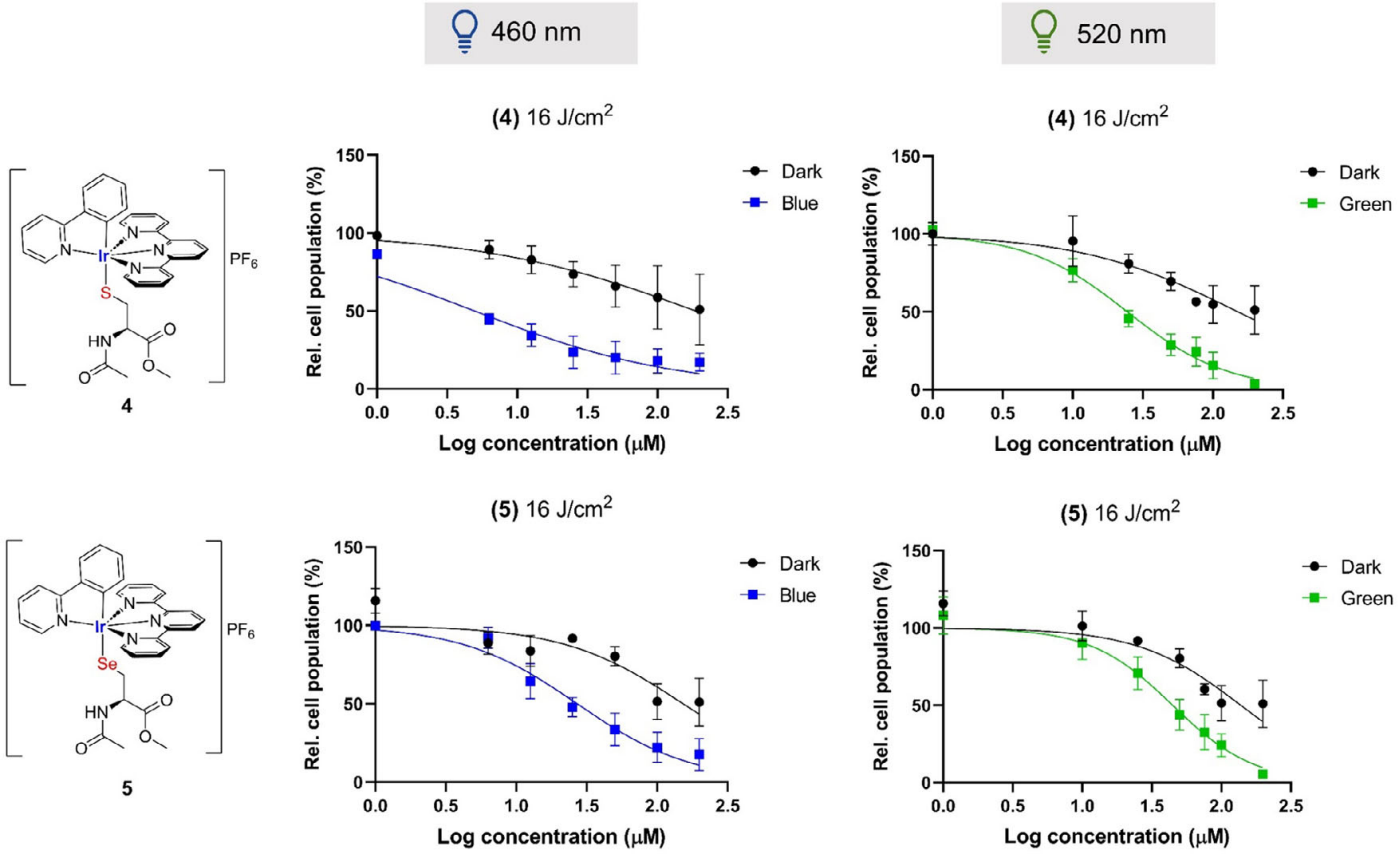
Dose‐response curves of A549 lung cancer cells exposed to **4** and **5** in the dark, after blue or green light illumination (16 J/cm^2^) under normoxia (21 % O_2_) determined with the MTT assay.

In photochemotherapy, light activation at longer wavelengths is often preferred due to higher tissue penetration depth and reduced photodamage to normal cells [48, 49]. To investigate the influence of the wavelength on the drug activation, the complexes were also illuminated with green light (520 nm, 16 J/cm^2^), matching the highest used in the blue light study. Although less effective than blue light, likely due to the lower extinction coefficients of the complexes at this wavelength, green light illumination still induced pronounced photocytotoxicity (Figure 8, right). Significant cell death was observed both with and without medium exchange prior to illumination (Table S6), indicating efficient cellular uptake and intracellular activation. Morphological analysis of A549 cells at 48 h after illumination revealed characteristic apoptotic features, including cell shrinkage and membrane blebbing (Figure S89). As chloride complex **1** is expected to be the major photoproduct of **4** or **5** in cells, its photocytotoxicity under blue light activation was also evaluated. In contrast to the caged complexes **4** and **5**, **1** showed moderate dark cytotoxicity (EC_50_, _dark_ = 36.7 µM), and phototoxicity upon blue‐light exposure (EC_50_, _light_ = 3.8 µM), suggesting efficient regeneration of ROS. Interestingly, under 16 J/cm^2^ blue light irradiation, the photocytotoxicity of **4** is like that of **1**, suggesting that upon efficient photorelease of the cysteinato ligand, PDT mediated toxicity dominates in the mode of action. No (photo)cytotoxicity was observed under hypoxic conditions (1% O_2_, Figures S87 and S88), consistent with the mechanistic studies, suggesting that dioxygen‐dependent Ir─S or Ir─Se bond cleavage is required to produce a PDT‐active photoproduct. Unlike in ruthenium‐based PACT, the specific O_2_‐dependent cleavage mode of the iridium‐chalcogenide bond makes the testing of the toxicity of the cysteine or selenocysteine ligands irrelevant, as the intact ligands are not released by photoactivation of **4** or **5** in cells, but rather the products of cysteine oxidation or Ir─Se bond cleavage. Overall, the cytotoxicity data suggest a biological mode of activation where the cysteinato or selenocysteinato ligands in **4** and **5** act as “photocaging” groups that require O_2_ for removal to produce the PDT sensitizer **1** or a solvent‐coordinated analogue.

### 2.6 Confocal Microscopy

Interestingly, both **1** and **6** are strongly phosphorescent whereas **4** and **5** are only weakly emissive. Thus, confocal microscopy was performed on A549 cells treated with **1**, **4**, or **5**, to check whether these differences in luminescence could also be observed in vitro. Unexpectedly, no discernible differences in luminescence were observed between cells kept in the dark under hypoxia prior to imaging and the illuminated cells under normoxia, indicating that the light used during confocal imaging was already sufficient to photoactivate **4** and **5** to generate an emissive photoproduct that could not be distinguished by its emissive properties from **1**. The only notable difference was the reduced number of cells in the plate illuminated prior to imaging, consistent with the photoinduced cytotoxicity measured in the MTT assay (Figure 9). Representative confocal microscopy images of A549 cells treated with **1**, **4**, or **5** are shown in Figure 9. Cells treated with **1** exhibited a higher background emission (Figure 9F,J), which is attributed to nonspecific binding of **1** and its higher emission quantum yield compared to **4**. Cells treated with **5** showed lower emission intensity than those with **4** and higher background emission (Figure 9H,L vs. G,K), which is consistent with the slower kinetic of activation of **5** compared with **4**. Overall, these results clearly indicated that **4** and **5** are much less emissive and PDT‐active than **1** in the dark, but that they transform, upon light exposure in presence of O_2_, into the emissive and PDT‐active photoproduct **1**. Although the intact complexes **4** and **5** could not be visualized in living cells, because the light source of the confocal microscope already triggered photoactivation, this result demonstrates their potential for highly localized photoactivation in biological tissue.

**FIGURE 9:**
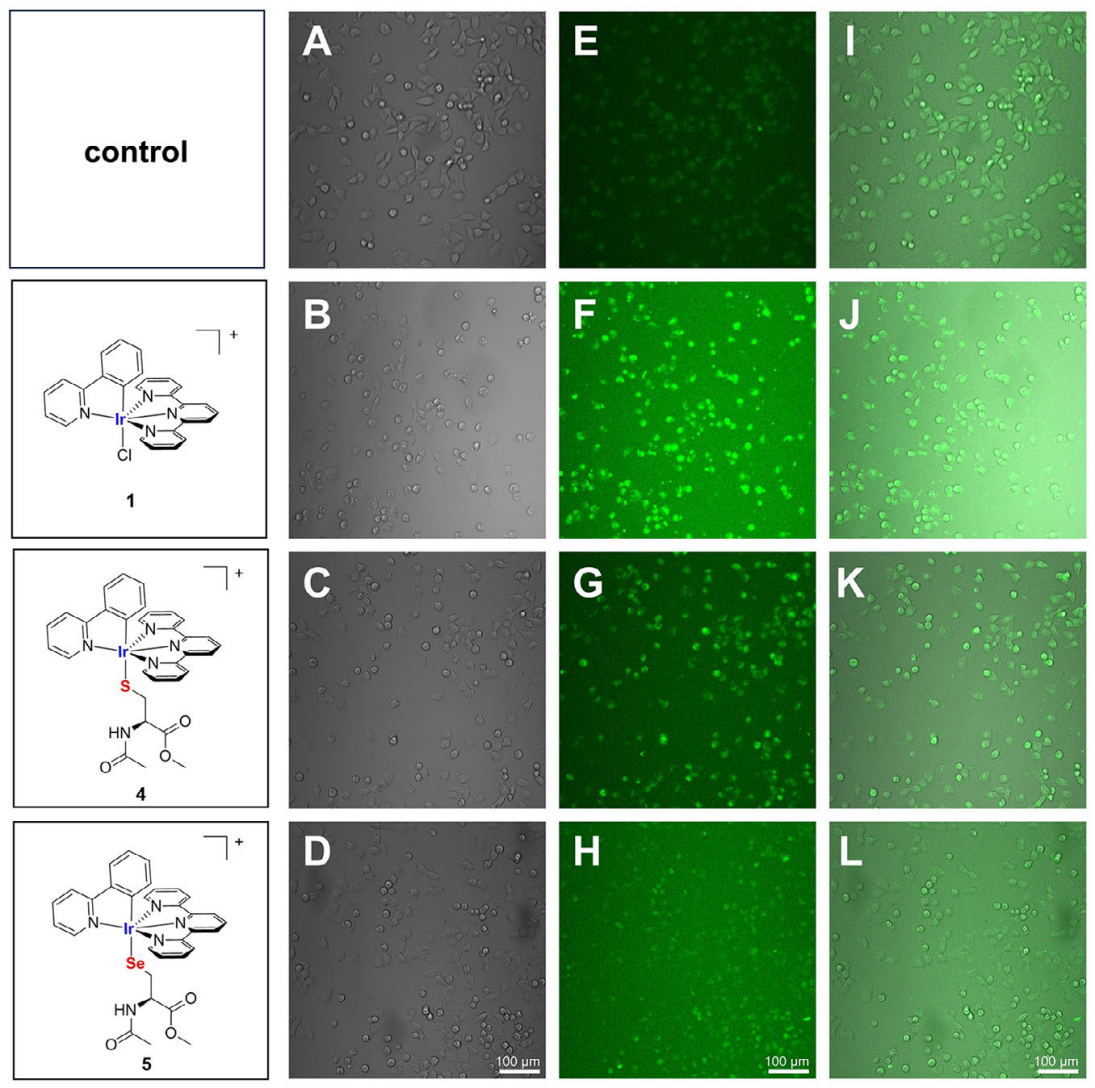
Confocal microscopy pictures of A549 lung cancer cells incubated with (40 µM) or without metal complexes for 8 h in the dark, followed by irradiation with 460 nm light (16 J/cm^2^) in normoxic conditions and further incubated for 17 h. Panels (A–D) show the brightfield, (E–H) show the green channel (luminescence of the iridium compounds, *λ*_ex/em_ = 405 nm, *λ*_em_ = 450–635 nm), and panels (I–L), the merged images of the brightfield and green channels. First row (A, E, I): control with no compound, showing some autofluorescence; second row (B, F, J): cells treated with [IrCl(ppy)(terpy)]PF_6_ (**1**); third row (C, G, K): cells treated with [Ir(cys)(ppy)(terpy)]PF_6_ (**4**); fourth row (D, H, L): cells treated with [Ir(sec)(ppy)(terpy)]PF_6_ (**5**).

## 3 Conclusion

Two new iridium(III) complexes of the general formula [Ir(L)(ppy)(terpy)]PF_6_ with L = cysteinato or selenocysteine were established as light‐activatable prodrugs for inorganic photochemotherapy. These compounds incorporate cysteine and selenocysteine derivatives as a “caging” group: the Ir─S and Ir─Se bonds exhibit high dark stability in various solvents and cell culture media but undergo efficient photocleavage by blue (460 nm) or green (515 nm) light in the presence of dioxygen, generating the phosphorescent and photodynamically active chlorido or solvate iridium complex. Both title complexes exhibit negligible singlet oxygen generation in their “caged” state but display high singlet oxygen quantum yields (40%–50%) after photoactivation, highlighting their potential as effective PDT type II photosensitizers. Further mechanistic investigations revealed distinct photoactivation pathways for the cysteinato versus selenocysteine complexes. In the cysteinato complex, initial oxidation of the iridium‐coordinated sulfur generates a sulfinato intermediate, which subsequently undergoes ligand exchange with the solvent (CH_3_CN) or a chloride ion, leading to the solvato **6** or chlorido complex **1** through a late stage Ir─S bond cleavage. In contrast, for the Se‐coordinated analogue, the identification of dehydroalanine by NMR spectroscopy and the first crystal structure of an iridium(III)‐selenito complex suggests an early‐stage photooxidative Se─C bond cleavage, leading to an iridium‐coordinated κ^O^‐selenito (Ir^III^‐SO_3_^2–^) intermediate. Although the nature of the intermediates is different, both title compounds ultimately convert to the same photoproduct, which features either a coordinated solvent molecule or chlorido as the monodentate ligand while the rest of the coordination sphere remains intact. This photoproduct is photodynamically active and hence capable of inducing phototoxicity upon further light activation, mostly by a type II PDT mechanism. Photocytotoxicity studies on A549 lung cancer cells demonstrated negligible dark toxicity (EC_50_ > 150 µM) for the title compounds, but potent photocytotoxicity upon blue or green light illumination, with PI values of up to 30. Generally, the cysteinato complex turned out to be more photoactive than the selenium analogue, mostly due to its higher Ir‐chalcogen bond‐cleavage photoreactivity. Overall, this work introduces a new class of iridium(III) prodrugs for combined PACT/PDT therapy, where a PACT bond cleavage photoreaction generates an iridium‐based sensitizer for PDT. These systems expand the scope of metal‐based photochemotherapy beyond well‐studied systems based on, for example, ruthenium and platinum. Future research will focus on optimizing the compounds towards red‐light activation for deeper tissue penetration and exploring additional toxic ligands to enhance the therapeutic efficacy via synergistic mechanisms.

## Author Contributions

**Victoria V. L. Müller**: methodology, investigation, validation, visualization, conceptualization, formal analysis, data curation, project administration. **Dominik Moreth**: writing – review and editing, investigation, data curation, methodology, validation, visualization, formal analysis. **Irene Regeni**: writing – review and editing, investigation, data curation, visualization, methodology. **Lan Wang**: investigation, data curation, writing – review and editing. **Sylvestre Bonnet**: funding acquisition, resources, writing – review and editing, project administration. **Ulrich Schatzschneider**: funding acquisition, resources, writing – review and editing, project administration.

## Conflicts of Interest

The authors declare no conflicts of interest.

## Supporting information




**Supporting File**: anie73852‐sup‐0001‐SuppMat.docx.

## Data Availability

The data that support the findings of this study are available in the Supporting Information of this article.
