# Supplementary material for: Uncaging Iridium(III) Complexes for Combined Photodynamic and Photoactivated Chemotherapy
Source: Angew Chem Int Ed Engl. 2026 Aug 12;65(41):e9954997. doi: 10.1002/anie.9954997 (PMC13638133; doi:10.1002/anie.9954997)
Supplement: Supplementary file 1 [file ANIE-65-e9954997-s001.docx]

**Supporting Information**

**Uncaging Iridium(III) Complexes for Combined Photodynamic and Photoactivated Chemotherapy**

Victoria V.L. Müller,^a,b*^ Dominik Moreth,^c,b^ Irene Regeni,^d,e^ Lan Wang,^d^ Sylvestre Bonnet,^d^ and Ulrich Schatzschneider^b^

*^a^* *Department of Chemistry, Princeton University, Princeton, NJ 08540,
United States of America*

*^b^* *Institut für Anorganische Chemie, Julius-Maximilians-Universität Würzburg,
Am Hubland, 97074 Würzburg, Germany*

*^c^ Faculty of Chemistry and Biochemistry, Ruhr-University Bochum, Universitätsstraße 150, 44801 Bochum, Germany*

*^d^ Leiden Institute of Chemistry, Universiteit Leiden, Einsteinweg 55, 2333 CC Leiden, The Netherlands*

*^e^ Institut de Science et d’Ingénierie Supramoléculaires (ISIS) University of Strasbourg & CNRS, 8 Allée, Gaspard Monge, 67000 Strasbourg, France*

* Corresponding author: vm2733@princeton.edu

Table of contents

[General remarks	3](#_Toc232674358)

[Synthesis of metal complexes	4](#_Toc232674359)

[Single-crystal X-ray diffraction analysis	17](#_Toc232674360)

[Dark stability of the metal complexes	22](#_Toc232674361)

[Photoactivation experiments monitored by UV/Vis and ESI mass spectrometry	23](#_Toc232674362)

[Fluorescence measurements	32](#_Toc232674363)

[Singlet oxygen quantum yield determination by phosphorescence	35](#_Toc232674364)

[Superoxide formation measurements	39](#_Toc232674365)

[Photoactivation of metal complexes monitored by NMR und ESI mass spectrometry	41](#_Toc232674366)

[Quantum Chemical calculations	49](#_Toc232674367)

[Photocytotoxicity in A549 cells	64](#_Toc232674368)

[References	70](#_Toc232674369)

# General remarks

All chemicals and solvents were purchased from Sigma-aldrich, abcr, or roth and used without further purification. [IrCl(ppy)(terpy)]PF_6_^1^ and *N*-acetyl-l-selenocysteine methyl ester^2^ were synthesized according to published procedures. Oxygen- and moisture-sensitive reactions were carried out using Schlenk techniques in oven-dried glassware under argon. Solvents were degassed by three freeze-pump-thaw cycles.

*Caution! Selenols are malodorous and potentially toxic. All reactions and handling should be carried out in a well-ventilated fume hood and waste has to be collected separately and properly disposed of according to local regulations.*

IR spectra of pure solid samples were collected on a *Jasco FT/IR-4100 spectrometer* fitted with a smart iTR ATR accessory in the range of 700–4000 cm^-1^ with 128 scans per sample. Signal intensities are reported as very strong (vs), strong (s), medium (m), weak (w), or broad (br). NMR spectra were recorded on a bruker Avance 400 spectrometer. Chemical shifts *δ* in ppm represent a downfield-shift relative to tetramethylsilane (TMS) and were referenced relative to the signal of the solvent.^3^ The ^77^Se NMR shift is reported relative to (H_3_C)_2_Se, respectively.^4^ Coupling constants *J* are given in Hertz (Hz). Peak multiplicities are indicated as singlet (s), doublet (d), triplet (t), doublet-of-doublet (dd), doublet-of-doublet-of-doublet (ddd), doublet-of-triplet (dt), triplet-of-doublet (td), and multiplet (m), respectively. Spectra were analysed with Bruker TopSpin (version 4.0.7). The regular mass spectra (ESI-MS) were recorded on a Shimadzu LCMS-2020 (ESI-Q). High resolution mass spectra were recorded on a ThermoFischer *Exactive Plus* spectrometer with an *Orbitrap* mass analyser and an ESI ion source (resolution *R* = 70000, sheath gas flow rate 15 L min^-1^, aux gas flow rate 5 L min^-1^, spray voltage 3.80 kV, capillary temperature 320 °C, S-lens level 50.0, and aux gas heater temperature 50 °C). The elemental composition of the compounds was determined with an Elementar Vario MICRO cube CHNS analyzer, Elementar UNICUBE CHNS analyzer or HEKAtech EURO EA CHNS analyzer. The addition of V_2_O_5_ was usually required to obtain proper results. UV/Vis spectra were recorded on an Agilent 8453 UV/Vis diode array spectrophotometer or a Agilent Cary 60 UV/Vis spectrometer. All data were analyzed and plotted using Origin Pro 2021b. Crystal data were collected on a Bruker X8-APEX II diffractometer with a CCD area detector using multi-layer mirror monochromated Mo_Kα_ radiation. Crystals were kept at 100(2) K during data collection.

# Synthesis of metal complexes

**Synthesis of [Ir(cys)(ppy)(terpy)]PF_6_ (4)**

[IrCl(ppy)(terpy)]PF_6_ (202 mg, 0.27 mmol) was dissolved in a mixture of degassed water and ethanol (1:3 *v*/v, 70 mL) at 60°C under argon. Then, solid *N*-acetyl-L-cysteine methyl ester (219 mg, 1.24 mmol) was added in portions, and the reaction mixture was stirred in the dark at 85°C for 22 h under argon. After cooling to room temperature, ammonium hexafluorophosphate (213 mg, 1.31 mmol) in water (2 mL) was added to the red solution and stirred for 10 minutes. The solvent was reduced to 4 mL under vacuum and. the resulting red precipitate filtered off, washed with cold water (3 × 3 mL), and dried under vacuum to give the product as a dark red solid. Yield: 49% (119 mg, 0.13 mmol). **IR** (ATR): 𝜈̃ = 3072 (w), 1742 (m), 1668 (w), 1609 (w), 1483 (w), 1453 (w), 839 (vs), 771 (s) cm^-1^; **^1^H NMR** (400.47 MHz, CD_3_CN): *δ* = 10.21 (ddd, 1H, ^3^*J*_H6,H5_ = 5.9 Hz, ^4^*J*_H6,H4_ = 1.7 Hz, ^5^*J*_H6,H3_ = 0.7 Hz, ppy-H6), 8.55 (d, 2H, ^3^*J*_H3’/H5’,H4’_ = 8.4 Hz, terpy-H3’/H5’), 8.40 (d, 2H, ^3^*J*_H3,H4/H3’’,H4’’_ = 8.1 Hz, terpy-H3/H3’’), 8.35 (t, 1H, ^3^*J* _H4‘,H3‘/H5‘_ *=* 8.2 Hz, terpy-H4’), 8.27 (d, 1H, ^3^*J*_H3,H4_ = 7.8 Hz, ppy-H3), 8.17 (ddd, 1H, ^3^*J*_H4,H5_ = 8.4 Hz, ^3^*J*_H4,H3_ = 7.4 Hz, ^4^*J*_H4,H6_ = 1.5 Hz, ppy-H4), 8.02 (dt, 2H, ^3^*J*_H4,H3/H5//H4’’,H3’’/H5’’_ = 8.0 Hz, ^4^*J*_H4,H6/H4’’,H6’’_ = 1.4 Hz, terpy-H4/H4’’), 7.82 (dd, 1H, ^3^*J*_H3’,H4‘_ = 7.8 Hz, ^4^*J*_H3’,H5‘_ = 1.2 Hz, ppy-H3’), 7.68-7.63 (m, 3H, terpy-H6/H6’’ and ppy-H5), 7.37 (ddd, 2H, ^3^*J*_H5,H4/H5‘‘,H4’’_ = 7.6 Hz, ^3^*J*_H5,H6/H5‘‘,H6’’_  = 5.7 Hz, ^4^*J*_H5,H3/H5’’,H3’’_ = 1.2 Hz, terpy-H5/H5’’), 6.96 (dt, 1H, ^3^*J*_H5‘,H4’/H6’_ = 7.6 Hz, ^4^*J*_H5’,H3’_ = 1.2 Hz, ppy-H5’), 6.77 (dt, 1H, ^3^*J*_H4’,H3’/H5’_ = 7.5 Hz, ^4^*J*_H4’,H6’_ = 1.2 Hz, ppy-H4’), 6.34 (d, 1H, ^3^*J*_NH,CH_ = 7.3 Hz, N*H*) 5.90 (dd, 1H, ^3^*J*_H6’,H5’_ = 7.7 Hz, ^5^*J*_H6’,H4’_ = 0.7 Hz, ppy-H6’), 3.74–3.78 (m, 1H, C*H*), 3.43 (s, 3H, OC*H*_3_), 1.84 (dd, 1H, ^2^*J* = 12.6 Hz, ^3^*J* = 6.3 Hz, C*H*_2_), 1.75 (s, 3H, C*H*_3_), 1.69 (dd, 1H, ^2^*J* = 12.6 Hz, ^3^*J* = 5.4 Hz, C*H*_2_) ppm; **^13^C NMR** (100.71 MHz, CD_3_CN): *δ* = 173.08 (*C*OOCH3), 170.55 (*C*OCH3), 169.00 (ppy-C2), 159.08 (terpy-C2/C2’’), 159.04 (terpy-C2/C2’’), 156.01 (terpy-C2’/C6’), 152.96 (ppy-C6), 152.90 (terpy-C6/C6’’),152.85 (terpy-C6/C6’’), 152.60 (ppy-C2’), 145.23 (ppy-C1’), 141.30 (ppy-C4), 140.80 (terpy-C4/C4’’), 139.91 (terpy-C4’), 132.02 (ppy-C6’), 131.92 (ppy-C4’), 130.00 (terpy-C5/C5’’), 126.90 (terpy-C3/C3’’),126.88 (terpy-C3/C3’’), 126.46 (ppy-C3’), 125.73 (ppy-C5), 125.69 (ppy-C5’), 125.58 (terpy-C3’/C5’), 125.87 (terpy-C3’/C5’), 122.19 (ppy-C3), 56.07 (*C*H), 52.81 (CO*C*H_3_), 27.34 (*C*H_2_), 23.27 (COO*C*H_3_) ppm; **MS** (ESI^+^, CH_3_OH) *m*/*z* = 756.1599 [M-PF_6_]^+^;
**Elemental analysis** (%): calcd. for C_32_H_29_F_6_IrN_5_O_3_PS (900.86 g mol^-1^): C 42.66, H 3.24, N 7.77, S 3.56; found (%): C 42.31, H 3.46, N 7.83, S 3.90.

The two outer terpy rings are not chemically equivalent in the ^13^C NMR. However, not all signals were observed due to overlaps caused by insufficient differences in chemical shift.

**Figure S1**: ATR IR spectrum of [Ir(cys)(ppy)(terpy)]PF_6_.

**Figure S2:** 400.47 MHz ^1^H NMR spectrum of [Ir(cys)(ppy)(terpy)]PF_6_ in CD_3_CN.

**Figure S3**: ^1^H,^1^H-NOESY spectrum of [Ir(cys)(ppy)(terpy)]PF_6_ in CD_3_CN.

**Figure S4**: 100.71 MHz ^13^C NMR spectrum of [Ir(cys)(ppy)(terpy)]PF_6_. in CD_3_CN.

**Figure S5**: Enlarged view of the ^13^C NMR spectrum of [Ir(cys)(ppy)(terpy)]PF_6_ in CD_3_CN.

**Figure S6**: ESI positive mode mass spectrum of [Ir(cys)(ppy)(terpy)]PF_6_ in methanol.

**Synthesis of [Ir(sec)(ppy)(terpy)]PF_6_ (5)**

[IrCl(ppy)(terpy)]PF_6_ (137 mg, 0.18 mmol) was dissolved in a mixture of degassed water and ethanol (1:3 v/v, 70 mL) at 60°C under argon. Then, solid N-acetyl-L-selenocysteine methyl ester (120 mg, 0.54 mmol) was added in portions and the reaction mixture was stirred under argon in the dark at 85°C for 22 h. After cooling to room temperature, ammonium hexafluorophosphate (108 mg, 0.66 mmol) in water (2 mL) was added to the brown solution and stirred for 10 min. The solvent was reduced to 3 mL under vacuum. The resulting brown precipitate was filtered off, washed with water (3 × 3 mL), and dried under vacuum to give the product as a brown solid. Yield: 51% (80.7 mg, 0.09 mmol). **IR** (ATR): 𝜈̃ = 3069 (w), 1739 (w), 1666 (w), 1607 (w), 1453 (w), 1212 (w), 1167 (w), 836 (vs), 765 (s) cm^-1^; **^1^H NMR** (400.47 MHz, CD_3_CN): *δ* = 10.32 (ddd, 1H, ^3^*J*_H6,H5_ = 5.8 Hz, ^4^*J*_H6,H4_ = 1.7 Hz, ^5^*J*_H6,H3_ = 0.7 Hz ppy-H6), 8.56 (d, 2H, ^3^*J*_H3’/H5’,H4’_ = 8.0 Hz, terpy-H3’/H5’), 8.40 (d, 2H, ^3^*J*_H3,H4/H3’’,H4’’_ = 8.1 Hz, terpy-H3/H3’’), 8.35 (t, 1H, ^3^*J* _H4‘,H3‘/H5‘_ *=* 8.2 Hz, terpy-H4’), 8.27 (d, 1H, ^3^*J*_H3,H4_ = 7.9 Hz, ppy-H3), 8.16 (dt, 1H, ^3^*J*_H4,H3/H5_ = 8.0 Hz, ^4^*J*_H4,H6_ = 1.5 Hz, ppy-H4), 8.01 (dt, 2H, ^3^*J*_H4,H3/H5//H4’’,H3’’/H5’’_ = 7.9 Hz, ^4^*J*_H4,H6/H4’’,H6’’_ = 1.3 Hz, terpy-H4/H4’’), 7.81 (dd, 1H, ^3^*J*_H3’,H4‘_ = 7.8 Hz, ^4^*J*_H3’,H5‘_ = 1.0 Hz, ppy-H3’), 7.71 (ddd, 2H, ^3^*J*_H6,H5/H6’’,H5’’_ = 5.6 Hz, ^4^*J*_H6,H4/H6’’,H4’’_ = 1.4 Hz, ^5^*J*_H6,H3/H6’’,H3’’_ = 0.7 Hz, terpy-H6/H6’’), 7.64 (ddd, 1H, ^3^*J*_H5,H6_ = 5.9 Hz, ^3^*J*_H5,H4_ = 7.3 Hz, ^4^*J*_H5,H3_ = 1.5 Hz, ppy-H5), 7.36 (ddd, 2H, ^3^*J*_H5,H4/H5‘‘,H4’’_ = 7.6 Hz, ^3^*J*_H5,H6/H5‘‘,H6’’_  = 5.7 Hz, ^4^*J*_H5,H3/H5’’,H3’’_ = 1.2 Hz, terpy-H5/H5’’), 6.96 (dt, 1H, ^3^*J*_H5‘,H4’/H6’_ = 7.6 Hz, ^4^*J*_H5’,H3’_ = 1.2 Hz, ppy-H5’), 6.77 (dt, 1H, ^3^*J*_H4’,H3’/H5’_ = 7.5 Hz, ^4^*J*_H4’,H6’_ = 1.2 Hz, ppy-H4’), 6.31 (d, 1H, ^3^*J*_NH,CH_ = 7.3 Hz, N*H*) 5.90 (d, 1H, ^3^*J*_H6’,H5’_ = 7.7 Hz, ^5^*J*_H6’,H4’_ = 0.7 Hz, ppy-H6’),3.74–3.78 (m, 1H, C*H*), 3.46 (s, 3H, COOC*H*_3_), 1.77–1.72 (m, 4H, COC*H*_3_ and C*H*_2_), 1.63 (dd, 1H, ^2^*J* = 11.8 Hz, ^3^*J* = 5.5 Hz, C*H*_2_) ppm; **^13^C NMR** (100.71 MHz, CD_3_CN): *δ* = 172.56 (*C*OOCH3), 170.01 (*C*OCH3), 168.32 (ppy-C2), 158.54, (terpy-C2/C2’’), 158.50 (terpy-C2/C2’’), 155.37 (terpy-C2’/C6’), 154.34 (ppy-C6), 152.57 (terpy-C6/C6’’), 152.52 (terpy-C6/C6’’), 151.28 (ppy-C1’), 144.66 (ppy-C2’), 140.75 (ppy-C4), 140.12 (terpy-C4/C4’’), 139.24 (terpy-C4’), 131.45 (ppy-C4’), 131.00 (ppy-C6’), 129.48 (terpy-C5/C5’’), 126.47 (terpy-C3/C3’’), 126.47 (terpy-C3/C3’’), 126.00 (ppy-C3’), 125.62 (ppy-C5), 125.37 (ppy-C5’), 125.16 (terpy-C3’/C5’), 121.81 (ppy-C3), 55.30 (*C*H), 52.39 (COO*C*H_3_), 22.79 (CO*C*H_3_), 13.45 (*C*H_2_) ppm; **^77^Se** **NMR** (76.38 MHz, CD3CN): δ = -59.98 ppm;

**MS** (ESI^+^, CH_3_OH) *m*/*z* = 804.1040 [M-PF_6_]^+^; **Elemental analysis** (%): calcd. for C_32_H_29_F_6_IrN_5_O_3_PSe (947.75 g mol^-1^): C 40.55, H 3.08, N 7.39; found (%): C 40.47, H 3.09, N 7.34.

The two outer terpy rings are not chemically equivalent in the ^13^C NMR. However, not all signals were observed due to overlaps caused by insufficient differences in chemical shift.

**Figure S7**: ATR IR spectrum of [Ir(sec)(ppy)(terpy)]PF_6_.

**Figure S8:** 400.47 MHz ^1^H NMR spectrum of [Ir(sec)(ppy)(terpy)]PF_6_ in CD_3_CN.

**Figure S9**: ^1^H,^1^H-NOESY spectrum of [Ir(sec)(ppy)(terpy)]PF_6_ in CD_3_CN.

**Figure S10**: 100.71 MHz ^13^C NMR spectrum of [Ir(cys)(ppy)(terpy)]PF_6_. in CD_3_CN.

**Figure S11:** Enlarged view of the ^13^C NMR spectrum of [Ir(sec)(ppy)(terpy)]PF_6_ in CD_3_CN.

**Figure S12:** 76.38 MHz ^77^Se NMR spectrum of [Ir(sec)(ppy)(terpy)]PF_6_ in CD_3_CN.

**Figure S13:** 76.38 MHz ^1^H-^77^Se HMQC NMR spectrum of of [Ir(sec)(ppy)(terpy)]PF_6_ in CD_3_CN.

**Figure S14**: ESI positive mode mass spectrum of [Ir(sec)(ppy)(terpy)]PF_6_ in methanol.

**Synthesis of [Ir(CH_3_CN)(ppy)(terpy)](PF_6_)_2_ (6)**

[IrCl(ppy)(terpy)]PF_6_ (50.4 mg, 0.07 mmol) and silver hexafluorophosphate (86.1 mg, 0.34 mmol) were dissolved in acetonitrile (20 mL). The reaction mixture was stirred in the dark at room temperature for 21 h. Then, the yellow reaction mixture was filtered through Celite, which was washed with acetonitrile (10 mL). The combined filtrates were collected, the solvent removed under reduced pressure, and the resulting material was dried under vacuum to give the product as a yellow solid. Yield: 78% (42.6 mg, 0.05 mmol). **^1^H NMR** (400.47 MHz, CD_3_CN): *δ* = 9.35 (ddd, 1H, ^3^*J*_H6,H5_ = 5.9 Hz, ^4^*J*_H6,H4_ = 1.9 Hz, ^5^*J*_H6,H3_ = 0.6 Hz, ppy-H6), 8.60 (d, 2H, ^3^*J*_H3’/H5’,H4’_ = 8.2 Hz, terpy-H3’/H5’), 8.51 (t, 1H, ^3^*J* _H4‘,H3‘/H5‘_ *=* 8.0 Hz, terpy-H4’), 8.44 (d, 2H, ^3^*J*_H3,H4/H3’’,H4’’_ = 8.2 Hz, terpy-H3/H3’’), 8.33 (d, 1H, ^3^*J*_H3,H4_ = 8.0 Hz, ppy-H3), 8.30-8.26 (m, 1H, ppy-H4), 8.17 (dt, 2H, ^3^*J*_H4,H3/H5//H4’’,H3’’/H5’’_ = 7.9 Hz, ^4^*J*_H4,H6/H4’’,H6’’_ = 1.6 Hz, terpy-H4/H4’’), 7.81 (dd, 1H, ^3^*J*_H3’,H4‘_ = 7.9 Hz, ^4^*J*_H3’,H5‘_ = 1.0 Hz, ppy-H3’), 7.75-7.70 (m, 3H, ppy-H5 and terpy-H6/H6’’), 7.48 (ddd, 2H, ^3^*J*_H5,H4/H5‘‘,H4’’_ = 7.7 Hz, ^3^*J*_H5,H6/H5‘‘,H6’’_  = 5.7 Hz, ^4^*J*_H5,H3/H5’’,H3’’_ = 1.4 Hz, terpy-H5/H5’’), 7.00 (dt, 1H, ^3^*J*_H5‘,H4’/H6’_ = 7.6 Hz, ^4^*J*_H5’,H3’_ = 1.2 Hz, ppy-H5’), 6.80 (dt, 1H, ^3^*J*_H4’,H3’/H5’_ = 7.5 Hz, ^4^*J*_H4’,H6’_ = 1.2 Hz, ppy-H4’), 5.90 (d, 1H, ^3^*J*_H6’,H5’_ = 7.8 Hz, ppy-H6’), 1.96 (s, 3H, C*H*_3_CN) ppm; **^13^C NMR** (100.69 MHz, CD_3_CN): *δ* = 166.66 (ppy-C2), 159.63 (terpy-C2/C2’’), 157.21 (terpy-C2’/C6’), 154.08 (terpy-C6/C6’’), 152.42 (ppy-C6), 145.46 (ppy-C2’), 143.15 (terpy-C4’), 142.85 (terpy-C4/C4’’), 142.36 (ppy-C4), 138.07 (ppy-C1’), 132.13 (ppy-C4’), 131.43 (ppy-C6’), 130.67 (terpy-C5/C5’’), 127.71 (terpy-C3/C3’’), 126.77 (ppy-C3’), 126.69 (ppy-C5), 126.57 (ppy-C5’), 126.43 (terpy-C3’/C5’), 122.86 (ppy-C3) ppm; **MS** (ESI^+^, CH_3_CN) *m*/*z* = 310.5745 [M-2PF_6_]^2+^; **Elemental analysis** (%): calcd. for C_28_H_22_F_12_IrN_5_P_2_ (910.66 g mol^-1^): C 36.93, H 2.44, N 7.69; found (%): C 34.69, H 2.43, N 7.00.

**Figure S15:** 400.47 MHz ^1^H NMR spectrum of [Ir(CH_3_CN)(ppy)(terpy)](PF_6_)_2_ in CD_3_CN.

**Figure S16:** 100.71 MHz ^13^C NMR spectrum of [Ir(CH_3_CN)(ppy)(terpy)](PF_6_)_2_ in CD_3_CN.

**Figure S17**: Enlarged view of the ^13^C NMR spectrum of [Ir(CH_3_CN)(ppy)(terpy)](PF_6_)_2_ in CD_3_CN.

**Figure S18**: ESI positive mode mass spectrum of [Ir(CH_3_CN)(ppy)(terpy)](PF_6_)_2_ in acetonitrile.

# Single-crystal X-ray diffraction analysis

Crystal data were collected on a Bruker X8-APEX II diffractometer with a CCD area detector using multi-layer mirror monochromated Mo_Kα_ radiation. Crystals were kept at 100(2) K during data collection. The structures were solved with ShelXT program^5^ using the intrinsic phasing method, refined with the ShelXL program,^6^ and expanded using Fourier techniques. All non-hydrogen atoms were refined anisotropically. Hydrogen atoms were included in structure factors calculations. All hydrogen atoms were assigned to idealised geometric positions. CCDC 2449061–2449064 contain the supplementary crystallographic data for this paper. These data can be obtained free of charge via www.ccdc.cam.ac.uk/data_request/cif, or by emailing data_request@ccdc.cam.ac.uk, or by contacting The Cambridge Crystallographic Data Centre, 12 Union Road, Cambridge CB2 1EZ, UK; fax: +44 1223 336033.

**Figure S19:** Molecular structure of [Ir(cys)(ppy)(terpy)]PF_6_ (**4**) with thermal displacement ellipsoids drawn at the 50% probability level. The hexafluorophosphate counterion and the solvent molecule are not shown for clarity.

**Table S1:** Crystallographic parameters for [Ir(cys)(ppy)(terpy)]PF_6._

| Data | [Ir(cys)(ppy)(terpy)]PF_6_ |
| --- | --- |
| Empirical formula | C_36_H_39_F_6_IrN_5_O_4_PS |
| Formula weight (g·mol^–1^) | 974.95 |
| Temperature (K) | 100(2) |
| Radiation, *λ* (Å) | Mo_Ka_, 0.71073 |
| Crystal system | monoclinic |
| Space group | *P*2_1_ |
| *Unit cell dimensions* |  |
| *a* (Å) | 8.630(6) |
| *b* (Å) | 15.464(9) |
| *c* (Å) | 14.310(8) |
| *α* (°) | 90 |
| *β* (°) | 103.383(17) |
| *γ* (°) | 90 |
| Volume (Å^3^) | 1858(2) |
| *Z* | 2 |
| Calculated density (Mg·m^–3^) | 1.743 |
| Absorption coefficient (mm^–1^) | 3.770 |
| *F*(000) | 968 |
| Theta range for collection | 1.463 to 26.500° |
| Reflections collected | 53142 |
| Unique reflections | 7622 |
| Unique reflections with [I>2σ(I)] | 5767 |
| Minimum/maximum transmission | 0.688/0.795 |
| Refinement method | Full-matrix least-squares on *F*^2^ |
| Data / parameters / restraints | 7622 / 491 / 1 |
| Goodness-of-fit on *F*^2^ | 1.02898 |
| Final *R* indices [*I*>2σ(*I*)] | *R_1_* = 0.0577, *wR_2_* = 0.1164 |
| *R* indices (all data) | *R_1_* = 0.0876, *wR_2_* = 0.1315 |
| Flack parameter | -0.017(12) |

**Figure S20:** Molecular structure of [Ir(sec)(ppy)(terpy)]PF_6_ (**5**) with thermal displacement ellipsoids drawn at the 50% probability level. The hexafluorophosphate counterion and solvent molecule are not shown for clarity.

**Table S2:** Crystallographic parameters for [Ir(sec)(ppy)(terpy)]PF_6._

| Data | [Ir(sec)(ppy)(terpy)]PF_6_ |
| --- | --- |
| Empirical formula | C_36_H_39_F_6_IrN_5_O_4_PSe |
| Formula weight (g·mol^–1^) | 1021.87 |
| Temperature (K) | 100(2) |
| Radiation, *λ* (Å) | Mo_Ka_, 0.71073 |
| Crystal system | monoclinic |
| Space group | *P*2_1_ |
| *Unit cell dimensions* |  |
| *a* (Å) | 8.688(6) |
| *b* (Å) | 15.601(12) |
| *c* (Å) | 14.264(8) |
| *α* (°) | 90 |
| *β* (°) | 104.04(3) |
| *γ* (°) | 90 |
| Volume (Å^3^) | 1875(2) |
| *Z* | 2 |
| Calculated density (Mg·m^–3^) | 1.809 |
| Absorption coefficient (mm^–1^) | 4.649 |
| *F*(000) | 1004 |
| Theta range for collection | 1.472 to 26.652° |
| Reflections collected | 57617 |
| Unique reflections | 7810 |
| Unique reflections with [I>2σ(I)] | 5564 |
| Minimum/maximum transmission | 0.541/0.640 |
| Refinement method | Full-matrix least-squares on *F*^2^ |
| Data / parameters / restraints | 7810 / 491 / 1 |
| Goodness-of-fit on *F*^2^ | 1.01792 |
| Final *R* indices [*I*>2σ(*I*)] | *R_1_* = 0.0589, *wR_2_* = 0.1290 |
| *R* indices (all data) | *R_1_* = 0.0958, *wR_2_* = 0.1512 |
| Flack parameter | -0.011(14) |

**Figure S21:** Molecular structure of [Ir(CH_3_CN)(ppy)(terpy)](PF_6_)_2_ (**6**) with thermal displacement ellipsoids drawn at the 50% probability level. Hexafluorophosphate counterions and solvent molecules not shown for clarity.

**Table S3:** Crystallographic parameters for [Ir(CH_3_CN)(ppy)(terpy)](PF_6_)_2._

| Data | [Ir(CH_3_CN)(ppy)(terpy)](PF_6_)_2_ |
| --- | --- |
| Empirical formula | C_32_H_28_F_12_IrN_7_P_2_ |
| Formula weight (g·mol^–1^) | 992.75 |
| Temperature (K) | 100(2) |
| Radiation, *λ* (Å) | Mo_Ka_, 0.71073 |
| Crystal system | monoclinic |
| Space group | *P*2_1_/*n* |
| *Unit cell dimensions* |  |
| *a* (Å) | 12.250(10) |
| *b* (Å) | 21.987(16) |
| *c* (Å) | 13.222(11) |
| *α* (°) | 90 |
| *β* (°) | 95.786(15) |
| *γ* (°) | 90 |
| Volume (Å^3^) | 3543(5) |
| *Z* | 4 |
| Calculated density (Mg·m^–3^) | 1.861 |
| Absorption coefficient (mm^–1^) | 3.958 |
| *F*(000) | 1936 |
| Theta range for collection | 1.804 to 31.676° |
| Reflections collected | 139289 |
| Unique reflections | 11842 |
| Unique reflections with [I>2σ(I)] | 8064 |
| Minimum/maximum transmission | 0.0077/0.0355 |
| Refinement method | Full-matrix least-squares on *F*^2^ |
| Data / parameters / restraints | 11842 / 519 / 66 |
| Goodness-of-fit on *F*^2^ | 1.018 |
| Final *R* indices [*I*>2σ(*I*)] | *R_1_* = 0.0469, *wR_2_* = 0.1041 |
| *R* indices (all data) | *R_1_* = 0.0814, *wR_2_* = 0.1208 |

**Figure S22**: Molecular structure of [Ir(SeO_3_)(ppy)(terpy)] (**8**) with thermal displacement ellipsoids drawn at the 50% probability level. The lattice-bound solvent molecule is not shown for clarity.

**Table S4**: Crystallographic parameters for [Ir(SeO_3_)(ppy)(terpy)].

| Data | [Ir(SeO_3_)(ppy)(terpy)] |
| --- | --- |
| Empirical formula | C_28_H_22_IrN_5_O_3_Se |
| Formula weight (g·mol^–1^) | 747.66 |
| Temperature (K) | 100(2) |
| Radiation, *λ* (Å) | Mo_Ka_, 0.71073 |
| Crystal system | orthorhombic |
| Space group | *Pbca* |
| *Unit cell dimensions* |  |
| *a* (Å) | 21.883(7) |
| *b* (Å) | 8.583(3) |
| *c* (Å) | 32.325(7) |
| *α* (°) | 90 |
| *β* (°) | 90 |
| *γ* (°) | 90 |
| Volume (Å^3^) | 6072(3) |
| *Z* | 8 |
| Calculated density (Mg·m^–3^) | 1.636 |
| Absorption coefficient (mm^–1^) | 5.632 |
| *F*(000) | 2880 |
| Theta range for collection | 1.861 to 28.547° |
| Reflections collected | 553352 |
| Unique reflections | 7693 |
| Unique reflections with [I>2σ(I)] | 5402 |
| Minimum/maximum transmission | .3606/0.5633 |
| Refinement method | Full-matrix least-squares on *F*^2^ |
| Data / parameters / restraints | 7693 / 344 / 0 |
| Goodness-of-fit on *F*^2^ | 1.036 |
| Final *R* indices [*I*>2σ(*I*)] | *R_1_* = 0.0630, *wR_2_* = 0.1750 |
| *R* indices (all data) | *R_1_* = 0.0900, *wR_2_* = 0.1955 |

# Dark stability of the metal complexes

**Figure S23**: Changes in the UV/Vis spectra of [Ir(cys)(ppy)(terpy)]PF_6_ (**4**) (left column) and [Ir(sec)(ppy)(terpy)]PF_6_ (**5**) (right column) in solution after incubation at room temperature for: 0 h (black trace), 24 h (red trace), and 48 h (blue trace). The solvent used was (top) acetonitrile, (center) water, and (bottom) Opti-MEM cell culture medium containing 1% DMSO.

**Figure S24**: ^1^H NMR spectra of [Ir(cys)(ppy)(terpy)]PF_6_ (**4**) in CD_3_CN/D_2_O (3:2, 6mM) upon incubation with excess glutathione (GSH) in the dark at different time points. The bottom spectrum shows free GSH as a reference.

# Photoactivation experiments monitored by UV/Vis and ESI mass spectrometry

All photoactivation experiments were performed with an Agilent Technologies Cary 60 UV-Vis spectrometer equipped with a Cary Single Cell Peltier Accessory for temperature control and a magnetic stirrer. Experiments were conducted in a quartz cuvette from Hellma Analytics (*d* = 1 cm) containing 3 mL of solution. A small LED (385 nm, 450 nm, 515 nm, 625 nm) was fitted on top of the cuvette and cooled by a water pump during the experiment. The surface power densities of the LEDs at the irradiation wavelength was measured using a Nova Power Meter from Ophir Photonics. All measurements were performed at 298 K under air in acetonitrile or water at a final concentration of 80 µM. The sample was continuously illuminated, and a spectrum recorded every 30 s for up to 90 min. After 90 min of illumination, the sample was analysed by ESI mass spectroscopy to identify the species present after light activation.

**Figure S25**: (**A**) Changes in the UV/Vis spectra of [Ir(cys)(ppy)(terpy)]PF_6_ (**4**) in acetonitrile under illumination at 385 nm (25.18 mW). The inset shows changes in the absorbance at 355  and 380 nm over the initial 5 min. (**B**) ESI mass spectrum of **4** in acetonitrile after illumination at 385 nm.

**Figure S26**: (**A**) Changes in the UV/Vis spectra of [Ir(cys)(ppy)(terpy)]PF_6_ (**4**) in acetonitrile under illumination at 450 nm (18.89 mW). The inset shows changes in the absorbance at 355 nd 380 nm over 20 min. (**B**) ESI mass spectrum of **4** in acetonitrile after illumination at 450 nm.

**Figure S27**: (**A**) Changes in the UV/Vis spectra of [Ir(cys)(ppy)(terpy)]PF_6_ (**4**) in acetonitrile under illumination at 515 nm (3.99 mW). The inset shows changes in the absorbance at 355 and 380 nm over 60 min. (**B**) ESI mass spectrum of **4** in acetonitrile after illumination at 515 nm.

**Figure S28**: Changes in the UV/Vis spectra of [Ir(cys)(ppy)(terpy)]PF_6_ (**4**) in acetonitrile under illumination at 625 nm (13.99 mW). The inset shows changes in the absorbance at 355 and 380 nm over 90 min. (**B**) ESI mass spectrum of **4** in acetonitrile after illumination at 625 nm.

**Figure S29:** (**A**) Changes in the UV/Vis spectra of [Ir(cys)(ppy)(terpy)]PF_6_ (**4**) in water under illumination at 385 nm (24.65 mW). The inset shows changes in the absorbance at 355 and 410 nm over 40 min. (**B**) ESI mass spectrum of **4** in water after illumination with 385 nm.

**Figure S30**: (**A**) Changes in the UV/Vis spectra of [Ir(cys)(ppy)(terpy)]PF_6_ (**4**) in water under illumination at 450 nm (19.13 mW). The inset shows changes in the absorbance at 355 and 410 nm over 90 min. (**B**) ESI mass spectrum of **4** in water after illumination at 450 nm.

**Figure S31**: (**A**) Changes in the UV/Vis spectra of [Ir(cys)(ppy)(terpy)]PF_6_ **4** in water under illumination at 515 nm (4.05 mW). The inset shows changes in the absorbance at 355 and 410 nm over 90 min. (**B**) ESI mass sepctrum of **4** in water after illumination at 515 nm.

**Figure S32**: (**A**) Changes in the UV/Vis spectra of [Ir(sec)(ppy)(terpy)]PF_6_ (**5**) in acetonitrile under illumination at 385 nm (23.09 mW). The inset shows changes in the absorbance at 355 and 380 nm over 60 min. (**B**) ESI mass spectrum of **5** in acetonitrile after illumination at 385 nm.

**Figure S33:** (**A**) Changes in the UV/Vis spectra of [Ir(sec)(ppy)(terpy)]PF_6_ (**5**) in acetonitrile under illumination at 450 nm (18.83 mW). The inset shows changes in the absorbance at 355 and 380 nm over 90 min. (**B**) ESI mass spectrum of **5** in acetonitrile after illumination at 450 nm.

**Figure S34**: (**A**) Changes in the UV/Vis spectra of [Ir(sec)(ppy)(terpy)]PF_6_ (**5**) in acetonitrile under illumination at 515 nm (3.66 mW). The inset shows changes in the absorbance at 355 and 380 nm over 90 min. (**B**) ESI mass spectrum of **5** in acetonitrile after illumination at 515 nm.

**Figure S35**: (**A**) Changes in the UV/Vis spectra of [Ir(sec)(ppy)(terpy)]PF_6_ (**5**) in acetonitrile under red light illumination (625 nm, 14.11 mW) for 90 min. (**B**) ESI mass spectrum of **5** in acetonitrile after illumination at 625 nm.

**Figure S36**: (**A**) Changes in the UV/Vis spectra of [Ir(sec)(ppy)(terpy)]PF_6_ (**5**) in water under illumination at 385 nm (24.03 mW). The inset shows changes in the absorbance at 355 and 380 nm over 90 min. (**B**) ESI mass spectrum of **5** in water after illumination at 385 nm.

**Figure S37**: (**A**) Changes in the UV/Vis spectra of [Ir(sec)(ppy)(terpy)]PF_6_ (**5**) in water under illumination at 450 nm (18.96 mW). The inset shows changes in the absorbance at 355 and 380 nm over 90 min. (**B**) ESI mass spectrum of **5** in water after illumination at 450 nm.

**Figure S38**: (**A**) Changes in the UV/Vis spectra of [Ir(sec)(ppy)(terpy)]PF_6_ (**5**) in water under illumination at 515 nm (3.81 mW). The inset shows changes in the absorbance at 355 and 380 nm over 90 min. (**B**) ESI mass spectrum of **5** in water after illumination at 515 nm.

# Fluorescence measurements

Florescence measurements were performed with a JASCO 60 FP-8300 spectrofluorometer equipped with a magnetic stirrer in quartz cuvettes from Hellma Analytics (*d* = 1 cm) containing 2 mL of solution. Illumination was carried out in the instrument, with a Xenon lamp at 385 nm (λ_exc_). All measurements were performed at 298 K under air in acetonitrile at a final concentration of 2–3 µM. For kinetic measurements, the sample was continuously illuminated, and a spectrum was recorded every 60 s for up to 180 min.

**Figure S39**: Stationary absorption and emission (*λ*_exc_ = 385 nm) spectra of [Ir(cys)(ppy)(terpy)]PF_6_ (**4**), [Ir(sec)(ppy)(terpy)]PF_6_ (**5**), and [Ir(CH_3_CN)(ppy)(terpy)](PF_6_)_2_ (**6**) in air-saturated acetonitrile at room temperature.

**Figure S40**: (**A**) Changes in the emission spectra of [Ir(cys)(ppy)(terpy)]PF_6_ (**4**) in air-saturated acetonitrile under 385 nm illumination. (**B**) Changes of the emission at 544 and 498 nm over 60 min.

**Figure S41**: (**A**) Changes in the emission spectra of [Ir(sec)(ppy)(terpy)]PF_6_ (**5**) in air-saturated acetonitrile under 385 nm illumination. (**B**) Changes of the emission at 544 and 498 nm over 60 min.

**Figure S42**: Changes in the emission spectrum of (A) [Ir(cys)(ppy)(terpy)]PF_6_ (**4**) and (B) [Ir(sec)(ppy)(terpy)]PF_6_ (**5**) at 60 µM in air-saturated OPTI-MEM cell culture medium; *λ*_exc_ = 405 nm) recorded with a microplate reader in a 96 well-plate under the same conditions as used in the cell illumination setup with increasing light exposure time (*λ*_illum_ = 460 nm, 19.17 mW/cm^2^).

**Relative Emission Quantum Yield**

Emission quantum yields were determined relative to [Ru(bpy)_3_]Cl_2_ in acetonitrile under air (*Φ*_reference_ = 0.018).^7^ Samples were prepared in acetonitrile and adjusted to an absorbance of ~0.1 at 380 nm. Emission spectra were recorded on a Jasco Spectrofluorometer FP-8300 using fluorescence quartz cuvettes (1 cm path length) with excitation at 380 nm. The relative quantum yields were calculated using the following equation:

$$\Phi_{sample}=\Phi_{reference}\times\left( \frac{A_{reference}}{A_{sample}} \right)\times\left( \frac{I_{sample}}{I_{reference}} \right)\times\left( \frac{n_{sample}}{n_{reference}} \right)^{2}$$

where:

*Φ*: emission quantum yield
*A*: absorbance at the excitation wavelength
*I*: integrated emission intensity
*n*: refractive index of the solvent

# Singlet oxygen quantum yield determination by phosphorescence

The steady-state singlet oxygen emission measurements were performed on a previously reported custom-built setup utilizing a slightly modified experimental procedure.^8-10^ Perinaphthenone was used as a reference which has a reported *Φ*_Δ_ (^1^O_2_) = 0.98 ± 0.07 in air-saturated acetonitrile.^11, 12^ [Ru(bpy)_3_]Cl_2_ was used as a validation compound with a reported *Φ*_Δ_ (^1^O_2_) = 0.57 ± 0.06 in air-saturated acetonitrile.^13^ All compounds were dissolved in acetonitrile (3 mL) to give solutions with an absorbance of *A* = 0.1 at the illumination wavelength and transferred to a macro-fluorescence cuvette from Firefly (light path: 1 × 1 cm). The illumination of samples was carried out at 298 K using a 450 nm LRD‐0450 Laserglow fiber‐coupled laser set to 80 mW at the cuvette with help of a PM100USB Thorlabs power meter. The NIR spectra were acquired within 20 s at 298 K with an Avantes NIR256‐1.7TEC spectrometer. To ensure that the laser pulse does not affect the light-sensitive samples, a UV/Vis spectrum of each compound was recorded before and after the measurement. All spectral data was processed with Origin 2021b and Microsoft Office Excel.

**Figure S43**:NIR emission spectra of ^1^O_2_ generated by perinaphthenone (black), [Ru(bpy)_3_]Cl_2_ (red), [IrCl(ppy)(terpy)]PF_6_ (**1**, cyan), [Ir(cys)(ppy)(terpy)]PF_6_ (4, blue), **4** after previous photoactivation (green), [Ir(sec)(ppy)(terpy)]PF_6_ (**5**, pink), and **5** after previous photoactivation (orange), all measured in air-saturated acetonitrile.

**Table S5**: Singlet oxygen quantum yields determined in acetonitrile.

| **Compound** | **Absorbance at 450 nm** | **Integrated NIR emission intensity** | ***Φ*_Δ_( ^1^O_2_)** |
| --- | --- | --- | --- |
| Perinaphthenone | 0.125 | 0.213 | 0.980 |
| [Ru(bpy)_3_]Cl_2_ | 0.117 | 0.122 | 0.604 |
| **1** | 0.110 | 0.098 | 0.519 |
| [Ir(cys)(ppy)(terpy)]PF_6_ (**4**) | 0.118 | 0.008 | 0.055 |
| **4** after photoactivation | 0.114 | 0.078 | 0.396 |
| [Ir(sec)(ppy)(terpy)]PF_6_ (**5**) | 0.101 | 0.000 | - |
| **5** after photoactivation | 0.111 | 0.092 | 0.481 |

**Figure S44**: NIR emission spectra emission from ^1^O_2_ generated by [Ir(cys)(ppy)(terpy)]PF_6_ (**4**) (blue) and **4** after photoactivation (green) in acetonitrile.

**Figure S45**: UV-Vis spectrum of [Ir(cys)(ppy)(terpy)]PF_6_ (**4**) in acetonitrile before (black) and after (red) singlet oxygen emission measurements under dark conditions. The changes in the absorbance spectra indicate that the sample already changed during the measurement.

**Figure S46**: NIR emission spectra emission from ^1^O_2_ by [Ir(sec)(ppy)(terpy)]PF_6_ (**5**) after photoactivation (orange spectrum) in acetonitrile. Under dark conditions, **5** produces no singlet oxygen and no emission is observed (pink spectrum).

**Figure S47**: UV-Vis spectrum of [Ir(sec)(ppy)(terpy)]PF_6_ (**5**) in acetonitrile before (black) and after (red) singlet oxygen emission measurements under dark conditions. No changes in the absorbance spectra indicate that the sample remained stable during the singlet oxygen measurement.

# Superoxide formation measurements

Superoxide generation by complexes 1, 4, and 5 in acetonitrile (80 µM) was investigated using dihydroethidium (DHE) as a chemoselective probe. DHE reacts with superoxide to form the emissive product 2-hydroxyethidium, which exhibits an emission band between 590 and 620 nm. Samples containing the iridium complex and DHE (200 µM) were irradiated with blue (450 nm) or green light (515 nm) for up to 20 min, and the emission intensity in the 590–620 nm region was monitored as a function of irradiation time.

**Figure S48**: Emission spectra of [IrCl(ppy)(terpy)]PF_6_ (**1**), [Ir(cys)(ppy)(terpy)]PF_6_ (**4**), [Ir(sec)(ppy)(terpy)]PF_6_ (**5**), and dihydroethidium (DHE) dissolved in acetonitrile/Opti-MEM) under blue light (top) or green light illumination (botttom) for 20 min.

# Photoactivation of metal complexes monitored by NMR und ESI mass spectrometry

**Figure S49**: ^1^H,^1^H-COSY spectrum of [Ir(cys)(ppy)(terpy)]PF_6_ (**4**) in CD_3_CN after 6 min of illumination with green light (520–525 nm). The cross peaks indicate correlation for the protons of the coordinated monodentate ligand.

**Figure S50:** ^1^H,^1^H-NOESY spectrum of [Ir(cys)(ppy)(terpy)]PF_6_ (**4**) in CD_3_CN after 6 min of illumination with green light (520–525 nm). The ppy-H6 peak of one species shows a cross peak with the CH_2_ group of the monodentate ligand.

**control**

**Figure S51**: ^1^H NMR spectra of [Ir(cys)(ppy)(terpy)]PF_6_ (**4**, 2.5 mM) and excess of tetrabutylammonium chloride (10 mM) in CD_3_CN after certain time intervalls irradtion with a green LED light (520-525 nm). The spectrum for [IrCl(ppy)(terpy)]PF_6_ (**1**) is as shown on top as a control.The species are assigned to **1** (green square), **4** (red dot), and an intermediate iridium species (blue triangle).

**Figure S 52**: ^1^H NMR spectra of [Ir(cys)(ppy)(terpy)]PF_6_ (**4**) in CD_3_CN under argon atmosphere after certain time intervalls illumination using a green LED light (520-525 nm).

**Figure S53**: Photograph of [Ir(cys)(ppy)(terpy)]PF_6_ (**4**) in CD_3_CN after illumination with green light for *t* = 4 min and *t* = 6 h under an argon atmosphere or under air. After 6 h total irradtion time, no further changes wee observed in the sample stored under argon, in contrast to the yellow solution of the sample exposed to air, which is characteristic for the solvent species [Ir(CH_3_CN)(ppy)(terpy)](PF_6_)_2_ (**6**).

**[M+O_2_-PF_6_]^+^**

**Figure S54**: Positive mode high-resolution ESI mass spectrum of a reaction mixture of [Ir(cys)(ppy)(terpy)]PF_6_ (**4**) in acetonitrile after 6 min of green light illumination.

**Calculated**

**[M+O_2_-PF_6_]^+^**

**Measured**

**Figure S55**: Enlarged view of the isotope pattern of the main signal at 788.1525 Da observed after 6 min of illumination of [Ir(cys)(ppy)(terpy)]PF_6_ (**4**) in acetonitrile with green light (blue trace) and simulated isotope pattern for [M+O_2_-PF_6_]^+^ (black trace).

**Calculated**

**[M-Cys-PF_6_+CH_3_CN]^2+^**

**Measured**

**Figure S56**: Enlarged view of the isotope pattern of the signal at 310.5745 Da observed after 6 min of illumination of [Ir(cys)(ppy)(terpy)]PF_6_ (**4**) in acetonitrile with green light (blue trace) and the simulated isotope pattern of [Ir(CH_3_CN)(ppy)(terpy)]^2+^ (black trace).

**Calculated**

**[M-Cys-PF_6_+Cl]^+^**

**Measured**

**Calculated**

**[M+O_2_-PF_6_]^+^**

**Measured**

**Figure S57**: Enlarged view of the isotope pattern of the signal at 615.0916 Da of observed after 6 min of illumination of [Ir(cys)(ppy)(terpy)]PF_6_ (**4**) in acetonitrile with green light (blue trace) and the simulated isotope pattern of [IrCl(ppy)(terpy)]^+^ (black trace).

**control**

**Figure S58**: ^1^H NMR spectra of [Ir(sec)(ppy)(terpy)]PF_6_ (**5**, 2.5 mM) in CD_3_CN after certain time intervalls illumination with a green LED light (520–525 nm). The spectrum for [Ir(CH_3_CN)(ppy)(terpy)](PF_6_)_2_ (**6**) is as shown on top as a reference.The species are assigned to **5** (red dot), an intermediate iridium species (blue triangle), and [Ir(CH_3_CN)(ppy)(terpy)]^2+^ (green squares).

**Figure S59**: Aliphatic region of the ^1^H NMR of [Ir(sec)(ppy)(terpy)]PF_6_ (**5**) after 60 min of green light illumination in CD_3_CN.

**control**

**Figure S60**: ^1^H NMR spectra of [Ir(sec)(ppy)(terpy)]PF_6_ (**5**, 2.5 mM) and excess of tetrabutylammonium chloride (10 mM) in CD_3_CN after certain time intervalls illumination with a green LED light (520–525 nm). The spectrum for [IrCl(ppy)(terpy)]PF_6_ **1** is as shown on top as reference. The species are assigned to **5** (red dot), **1** (green square), and [Ir(CH_3_CN)(ppy)(terpy)]^2+^ (signal at 9.35 ppm).

**Figure S61**: ^1^H NMR spectra of [Ir(sec)(ppy)(terpy)]PF_6_ (**5**) in CD_3_CN under argon atmosphere after certain time intervalls illumination using a green LED light (520–525 nm).

**Figure S62**: Positive mode high-resolution ESI mass spectrum of a reaction mixture of [Ir(sec)(ppy)(terpy)]PF_6_ (**5**) in acetonitrile after 20 min of green light illumination.

**Calculated**

**[M-C_6_H_9_NO_3_+O_2_+OH-PF_6_]^+^**

**Measured**

**Figure S63**: Enlarged view of the isotope pattern of the signal at 709.0315 Da of [Ir(sec)(ppy)(terpy)]PF_6_ (**5**) in acetonitrile after 20 min of illumination with green light (blue trace) and the simulated isotope pattern of [Ir(HSeO_3_)(ppy)(terpy)]^+^ (black trace).

# Quantum Chemical calculations

Density functional theory (DFT) and time-dependent DFT (TD-DFT) calculations were performed using the ORCA program package (version 6.0.1).^[1]^ All calculations employed the B3LYP hybrid functional in combination with Ahlrichs’ polarized triple-zeta basis set (def2-TZVP) and the SARC/J auxiliary basis set.^[2,3]^ Relativistic effects for Iridium were treated using the zeroth-order regular approximation (ZORA) with appropriately recontracted relativistic basis sets.^[4]^ Tight SCF convergence criteria were applied throughout (TightSCF). Solvent effects were modelled using the conductor-like polarizable continuum model (CPCM) with water as solvent. Grimme’s D4 dispersion correction was included in all calculations.^[5]^ Explicit solvent molecules or counterions were not considered. Geometry optimizations were performed for the ground state (S₀). All optimized structures were confirmed as local minima via harmonic vibrational frequency analysis (no imaginary frequencies). Hirshfeld population analysis and Mayer bond order analysis were additionally performed to evaluate charge distribution and metal–ligand bonding characteristics within the complexes. Spin–orbit coupled TDDFT (SOC-TDDFT) calculations were carried out without the Tamm–Dancoff approximation (TDA) to compute the lowest 50 singlet and triplet excited states (NROOTS 25). Charge transfer number analysis of the excited states was performed using TheoDORE 3.2.^[6]^ The complex cation was divided into four fragments: (i) the Ir center, (ii) the terpyridine ligand, (iii) the phenylpyridine ligand, and (iv) the monodentate ligand (X = Cl, Cys, Sec, CysO_2_, SecO_2_, SeO_3,_ ACN). Molecular orbitals were visualized using the open-source molecular builders and visualization tools Avogadro (version 1.2.0) and Chemcraft (version 1.8).

**Figure S 64:** Optimized geometry of **1** in ground state (S_0_).

**Table S 6:** Comparison of experimental and theoretically calculated structural and energetic parameters of 1: SCF Energies (Eh), Bond Lengths (Å), and Bond Angles (°)

|  | Exp.  SC-XRD | Calc.  S_0_ |
| --- | --- | --- |
| SCF-Energy |  | -20100.382487478317 |
|  |  |  |
| Ir1-C1 | 2.026(10) | 2.014 |
| Ir1-N1 | 2.074(9) | 2.081 |
| Ir1-N2 | 2.039(9) | 2.053 |
| Ir1-N3 | 1.936(9) | 1.960 |
| Ir1-N4 | 2.041(9) | 2.052 |
| Ir1-Cl1 | 2.427(3) | 2.513 |
| C1-Ir1-N2 | 93.7(4) | 91.1 |
| C1-Ir1-N3 | 94.9(4) | 96.6 |
| N1-Ir-N2 | 99.1(4) | 99.7 |
| N1-Ir1-Cl1 | 94.5(3) | 95.6 |
| N2-Ir-Cl1 | 89.2(2) | 89.5 |
| N3-Ir-Cl1 | 90.3(2) | 87.7 |
| N4-Ir-Cl1 | 88.6(2) | 89.7 |

**Figure S 65:** TDDFT electron difference densities between S_0_ and T_1_ (isosurface value 0.002, orange: electron loss, green: electron gain) of **1**.

**Figure S 66:** Charge transfer number analysis for **1** from TDDFT calculations a) with iridium, ppy, terpy and chloride as fragments.

**Figure S 67:** Optimized geometry of **4** in ground state (S_0_).

**Table S 7:** Comparison of experimental and theoretically calculated structural and energetic parameters of **4**: SCF Energies (Eh), Bond Lengths (Å), and Bond Angles (°)

|  | Exp.  SC-XRD | Calc.  S_0_ |
| --- | --- | --- |
| SCF-Energy |  | -20553.395816864682 |
|  |  |  |
| Ir1-C1 | 2.039(18) | 2.041 |
| Ir1-N1 | 2.090(14) | 2.093 |
| Ir1-N2 | 2.038(13) | 2.053 |
| Ir1-N3 | 1.955(13) | 1.953 |
| Ir1-N4 | 2.014(14) | 2.050 |
| Ir1-S1 | 2.443(5) | 2.481 |
| S1-C27 | 1.800(17) | 1.832 |
| C1-Ir1-N2 | 90.3(6) | 90.2 |
| C1-Ir1-N3 | 95.2(8) | 96.4 |
| N1-Ir1-N2 | 101.8(5) | 100.0 |
| N1-Ir1-S1 | 96.1(4) | 95.1 |
| N2-Ir1-S1 | 89.1(4) | 89.1(4) |
| N3-Ir1-S1 | 88.8(6) | 88.8(6) |
| N4-Ir1-S1 | 90.6(4) | 90.6(4) |
| Ir1-S1-C27 | 105.3(6) | 105.6 |

**Figure S 68:** TDDFT electron difference densities between S_0_ and T_1_ (isosurface value 0.002, orange: electron loss, green: electron gain) of **4**.

**Figure S 69:** Charge transfer number analyses for **4** from TDDFT calculations a) with iridium, ppy, terpy and SPh as fragments.

**Figure S 70:** Optimized geometry of **5** in ground state (S_0_).

**Table S 8:** Comparison of experimental and theoretically calculated structural and energetic parameters of **5**: SCF Energies (Eh), Bond Lengths (Å), and Bond Angles (°)

|  | Exp.  SC-XRD | Calc.  S_0_ |
| --- | --- | --- |
| SCF-Energy |  | -22599.294686239511 |
|  |  |  |
| Ir1-C1 | 2.03(2) | 2.039 |
| Ir1-N1 | 2.068(15) | 2.095 |
| Ir1-N2 | 2.050(15) | 2.050 |
| Ir1-N3 | 1.945(13) | 1.953 |
| Ir1-N4 | 2.043(16) | 2.053 |
| Ir1-Se1 | 2.560(3) | 2.613 |
| Se1-C27 | 1.954(18) | 1.987 |
| C1-Ir1-N2 | 91.5(7) | 90.3 |
| C1-Ir1-N3 | 96.5(8) | 96.2 |
| N1-Ir1-N2 | 97.1(6) | 100.0 |
| N1-Ir1-Se1 | 97.0(4) | 96.0 |
| N2-Ir1-Se1 | 89.7(4) | 87.6 |
| N3-Ir1-Se1 | 87.2(5) | 88.3 |
| N4-Ir1-Se1 | 88.6(4) | 92.4 |
| Ir1-Se1-C27 | 102.7(6) | 102.0 |

**Figure S 71:** TDDFT electron difference densities between S_0_ and T_1_ (isosurface value 0.002, orange: electron loss, green: electron gain) of **5**.

**Figure S 72:** Charge transfer number analyses for **5** from TDDFT calculations a) with iridium, ppy, terpy and SePh as fragments.

**Figure S 73:** Optimized geometry of **7** in ground state (S_0_).

**Table S 9:** Comparison of experimental and theoretically calculated structural and energetic parameters of **7**: SCF Energies (Eh), Bond Lengths (Å), and Bond Angles (°)

|  | Calc.  S_0_ |
| --- | --- |
| SCF-Energy | -20704.027991571478 |
|  |  |
| Ir1-C1 | 2.047 |
| Ir1-N1 | 2.096 |
| Ir1-N2 | 2.057 |
| Ir1-N3 | 1.965 |
| Ir1-N4 | 2.061 |
| Ir1-S1 | 2.426 |
| S1-O1 | 1.490 |
| S1-O2 | 1.485 |
| S1-C27 | 1.826 |
| C1-Ir1-N2 | 89.2 |
| C1-Ir1-N3 | 94.5 |
| N1-Ir1-S1 | 97.7 |
| N2-Ir1-S1 | 93.5 |
| N3-Ir1-S1 | 88.2 |
| N4-Ir1-S1 | 93.5 |
| O1-S1-O2 | 112.7 |
| Ir1-S1-C27 | 110.8 |

**Figure S 74:** TDDFT electron difference densities between S_0_ and T_1_ (isosurface value 0.002, orange: electron loss, green: electron gain) of **7**.

**Figure S 75:** Charge transfer number analyses for **7** from TDDFT calculations a) with iridium, ppy, terpy and SePh as fragments.

**Figure S 76:** Optimized geometry of **8** in ground state (S_0_).

**Table S 10:** Comparison of experimental and theoretically calculated structural and energetic parameters of **8**: SCF Energies (Eh), Bond Lengths (Å), and Bond Angles (°)

|  | Calc.  S_0_ |
| --- | --- |
| SCF-Energy | -22749.870014699132 |
|  |  |
| Ir1-C1 | 2.031 |
| Ir1-N1 | 2.088 |
| Ir1-N2 | 2.061 |
| Ir1-N3 | 1.970 |
| Ir1-N4 | 2.060 |
| Ir1-Se1 | 2.580 |
| Se1-O1 | 1.672 |
| Se1-O2 | 1.662 |
| Se1-C27 | 1.982 |
| C1-Ir1-N2 | 89.5 |
| C1-Ir1-N3 | 94.7 |
| N1-Ir1-Se1 | 98.7 |
| N2-Ir1-Se1 | 92.2 |
| N3-Ir1-Se1 | 86.8 |
| N4-Ir1-Se1 | 87.4 |
| O1-Se1-O2 | 110.0 |
| Ir1-Se1-C27 | 112.6 |

**Figure S 77:** TDDFT electron difference densities between S_0_ and T_1_ (isosurface value 0.002, orange: electron loss, green: electron gain) of **8.**

**Figure S 78:** Charge transfer number analyses for **8** from TDDFT calculations a) with iridium, ppy, terpy and SeO2Ph as fragments.

**Figure S 79:** Optimized geometry of **9** in ground state (S_0_).

**Table S 11:** Comparison of experimental and theoretically calculated structural and energetic parameters of **9**: SCF Energies (eV), Bond Lengths (Å), and Bond Angles (°)

|  | Exp.  SC-XRD | Calc.  S_0_ |
| --- | --- | --- |
| SCF-Energy |  | -22309.777583401417 |
|  |  |  |
| Ir1-C1 | 2.013(10) | 2.022 |
| Ir1-N1 | 2.070(8) | 2.076 |
| Ir1-N2 | 2.053(8) | 2.048 |
| Ir1-N3 | 1.946(8) | 1.956 |
| Ir1-N4 | 2.036(8) | 2.048 |
| Ir1-O1 | 2.142(7) | 2.143 |
| Se1-O1 | 1.665(7) | 1.761 |
| Se1-O2 | 1.883(11) | 1.682 |
| Se1-O3 | 1.606(8) | 1.682 |
| N1-Ir1-N2 | 97.5(3) | 99.6 |
| N1-Ir1-O1 | 88.8(3) | 88.0 |
| N2-Ir1-O1 | 93.2(3) | 90.8 |
| N3-Ir1-O1 | 96.8(3) | 97.3 |
| N4-Ir1-O1 | 86.0(3) | 90.8 |
| O1-Se1-O2 | 99.5(4) | 103.8 |
| O2-Se1-O3 | 93.7(5) | 103.9 |
| O1-Se1-O3 | 108.2(4) | 103.9 |

**Figure S 80:** TDDFT electron difference densities between S_0_ and T_1_ (isosurface value 0.002, orange: electron loss, green: electron gain) of **9.**

**Figure S 81:** Charge transfer number analyses for **9** from TDDFT calculations a) with iridium, ppy, terpy and SeO2Ph as fragments.

**Figure S 82:** Optimized geometry of **6** in ground state (S_0_).

**Table S 12:** Comparison of experimental and theoretically calculated structural and energetic parameters of **6**: SCF Energies (eV), Bond Lengths (Å), and Bond Angles (°)

|  | Exp.  SC-XRD | Calc.  S_0_ |
| --- | --- | --- |
| SCF-Energy |  | -537986.41735 |
|  |  |  |
| Ir1-C1 | 2.012(5) | 2.015 |
| Ir1-N1 | 2.068(4) | 2.077 |
| Ir1-N2 | 2.049(4) | 2.061 |
| Ir1-N3 | 1.974(4) | 1.970 |
| Ir1-N4 | 2.056(4) | 2.060 |
| Ir1-N5 | 2.134(4) | 2.128 |
| C1-Ir1-N2 | 90.18(18) | 90.6 |
| C1-Ir1-N3 | 93.43(18) | 95.4 |
| N1-Ir-N2 | 79.80(17) | 80.2 |
| N1-Ir1-N5 | 91.00(15) | 89.9 |
| N2-Ir-N5 | 94.05(17) | 90.3 |
| N3-Ir-N5 | 95.62(15) | 95.6 |
| N4-Ir-N5 | 86.79(16) | 88.7 |

**Figure S 83:** TDDFT electron difference densities between S_0_ and T_1_ (isosurface value 0.002, orange: electron loss, green: electron gain) of **6**.

**Figure S 84:** Charge transfer number analysis for **6** from TDDFT calculations a) with iridium, ppy, terpy and chloride as fragments.

# Photocytotoxicity in A549 cells

**Cell culturing**

Human cancer cell line A549 (human lung carcinoma, RRID: CVCL_0023) was purchased from Merck. Cells were thawed and passaged at least twice before starting cytotoxicity experiments. Cells were cultured in Dulbecco’s Modified Eagle Medium with phenol red, supplemented with 10.0% *v*/*v* fetal calf serum (FCS), 0.2% *v*/*v* penicillin/streptomycin (P/S), and 1% *v*/*v* Glutamine-S (GM) (DMEM complete) The cells were incubated in the dark at 37 °C, 21% O_2_, and 5% CO_2_ in 75 cm^2^ flasks and passaged at 70–80% confluence. Cells used in all biological experiments were cultured for up to eight weeks. DMEM (with phenol red) and Opti-MEM® (without phenol red, with L-Glutamine) were purchased from Gibco® Life Technologies. PBS buffer was purchased from Sigma Aldrich. Cells were inspected with an Olympus IX81 microscope. Trypan blue (0.4% in 0.81% sodium chloride and 0.06% potassium phosphate dibasic solution) was purchased from BioRad. Cells were counted using a BioRad TC10 automated cell counter with BioRad Cell Counting Slides. UV-Vis measurements for analysis of 96-well plates were performed on an Infinite® M1000 Tecan Reader.

**Photocytotoxicty assay of A549 cells**

The photocytotoxicity of A549 cancer cells was assessed using the assay described by Hopkins et al.^14^ For each photocytoxicity experiment, an additional identical control plate was prepared to serve as the dark control. A549 cells were seeded at *t* = 0 h in 96-well plates (Greiner) at a density of 5.000 cells/well (100 µL) in OPTI-MEM (Gibco complete medium 11058-021), supplemented with 2.5% *v*/*v* fetal calf serum (FCS), 0.2% *v*/*v* penicillin/streptomycin (P/S), and 1% *v*/*v* glutamine. The cells were incubated for 24 h. To prevent border effects, only the inner 60 wells were used for seeding, while the outer wells were filled with OPTI-MEM. For preparation of stock solutions, the metal complexes were dissolved in sterilized DMSO and diluted with OPTI-MEM to a 1 mM stock solution, followed by serial dilutions with OPTI-MEM to obtain aliquots of different concentrations. After 24 hours of incubation, 100 µL of the freshly prepared aliquots were added to the wells in triplicate, reaching a total medium volume of 200 µL in each well. The final DMSO concentration per well did not exceed 0.5% and had no effect on cell viability. An aqueous cisplatin solution was prepared from a stock solution based on clinical formulation (3.3 mM cisplatin, 55 mM mannitol, 154 mM NaCl) and was always used as a positive control (*n* = 6).^15^ Plates were incubated in the dark at 37 °C (21% O_2_ and 7.0% CO_2_) for 24 h. In the standard protocol, at *t* = 48 h, one plate was illuminated with green light (520 nm) for 30 min at 37 °C (dose: 16 J/cm^2^, intensity: 9.04 mW/cm^2^), blue light (460 nm) for 7 min at 37 °C (dose: 8 J/cm^2^, intensity: 19.2 mW/cm^2^) or 14 min at 37 °C (dose: 16 J/cm^2^, intensity: 19.2 mW/cm^2^), while the other plate was kept in the dark as the control. In a second set of experiments, the protocol was modified by replacing the medium with drug-free fresh medium before irradiation. In both protocols, the cells were further incubated for another 48 hours in the dark. Finally, at *t* = 96 h, relative cell viability was determined by a colorimetric MTT end-point assay.

**MTT assay**

An MTT (3-(4,5-dimethylthiazol-2-yl)-2,5-diphenyltetrazolium bromide) solution was prepared by dissolving 5 mg/mL MTT (yellow solid) in PBS at least 1 h before use. At *t* = 96 h, 10 µL of this MTT solution was added to each well, and the plate was incubated for 3–4 h at 37 °C (21% O_2_ and 7.0% CO_2_). The solvent was carefully removed, and the purple formazan crystals were then solubilized in 200 µL DMSO (≥ 99.0%, VWR chemicals 23486.322), and the absorbance was measured at 570 nm using a Tecan plate reader. The absorbance data per compound per concentration were averaged over three identical wells (technical replicates, *n* = 3). Relative cell populations were calculated by dividing the average value of the treated cells by the average of the untreated control cells (*n* = 12) in Excel. All experiments were conducted in three independent biological replicates, and the obtained data were analysed with GraphPad Prism 9. The EC_50_ concentration (defined as the concentration of drug that kills 50% of cells compared to the untreated control) was calculated using the non-linear two-parameter Hill slope equation with a fixed maximum (100 %) and minimum (0%).^14^

$$Y=\frac{100}{(1+{10}^{\left( \left( {log}_{10}{EC}_{50}-X \right)\times Hill slope \right)})}$$

**Table S13**: EC*_50_* values for compounds **4** and **5** in A549 cells with medium exchange to drug-free medium before light activation.

| Compound | Dark | | Blue (460 nm) | | | | | | Green (520 nm) | | |
| --- | --- | --- | --- | --- | --- | --- | --- | --- | --- | --- | --- |
|  |  |  | 8 J/cm^2^ | | | 16 J/cm^2^ | | | 16 J/cm^2^ | | |
|  | EC_50_  (μM) | CI ^a)^  95%  (μM) | EC_50_  (μM) | CI ^a)^  95%  (μM) | PI | EC_50_  (μM) | CI ^a)^  95%  (μM) | PI | EC_50_  (μM) | CI ^a)^  95%  (μM) | PI |
| **4** | >200 | - | 21.2 | -4.2 +5.1 | >9.4 | 9.7 | -2.4 +2.7 | >20.5 | 35.9 | -4.4 +4.7 | >5,7 |
| **5** | >200 | - | 61.1 | -9.7 +12.3 | >3.3 | 34.4 | -7.0 +9.4 | >5.8 | 71.1 | -7.5 +8.6 | >2.8 |

^a)^ 95% Confidence Interval based on biologically independent triplicates (n=3).

**(5)**

**(5)**

**(4)**

**(5)**

**(4)**

**(4)**

**Figure S85**: Dose-response curves for (left column) **4** and (right column) **5** in A549 cells with medium exchange in the dark and upon blue light illumination with a lightdose of 8 J/cm^2^ (top row) or 16 J/cm^2^ (middle row), and green light illumination with 16 J/cm^2^ (bottom row).

**(5)**

**(4)**

**(4)**

**(4)**

**(5)**

**(5)**

**Figure S86**: Dose-response curves for (left column) **4** and (right column) **5** in A549 cells determined with the standard protocoll (without medium exchange) in the dark and upon blue light illumination with a lightdose of 8 J/cm^2^ (top row) or 16 J/cm^2^ (middle row) and green light illumination with 16 J/cm^2^ (bottom row).

**Figure S87:** Dose-response curves for (left) **4** and (right) **5** in A549 cells determined with the standard protocoll (without medium exchange) in the dark and upon blue and green light illumination with a lightdose of 16 J/cm^2^ (blue squares) and green light illumination with 16 J/cm^2^ (green squares) in hypoxia (1% O_2_).

**Figure S88:** Dose-response curves for (left) **4** and (right) **5** in A549 cells with medium exchange in the dark and upon blue and green light illumination with a lightdose of 16 J/cm^2^ (blue squares) and green light illumination with 16 J/cm^2^ (green squares) in hypoxia (1% O_2_).

**Figure S89**: Bright field microscope images of A549 cells treated with **4** (25 μM) in the dark, photoactivated **4** (25 μM), and cisplatin. The medium was exchanged before illumination with blue light (460 nm, 16 J/cm^2^). At 48 h after illumination, the cell morphology became similar to the one observed with cisplatin incubation.

**Confocal Microscopy**

Confocal imaging was performed using an Eclipse Ti2-C2+ Nikon confocal microscope equipped with a 40× water objective (Apo LWD 40×/1.15 WI λS DIC N2). Luminescence from the tested compounds was excited using a 405 nm laser, and emission was collected in the 450–635 nm range. The imaging chamber was maintained at 37 °C with an oxygen concentration of 1% to prevent unintended activation of metal complexes **4** and **5**. Plate irradiation was carried out as described in the cytotoxicity assay section. Image acquisition settings (laser power, PMT gain, and pinhole size) were kept constant across all experimental conditions. Images were processed using NIS-Elements AR (v5.02.02), Fiji (ImageJ), and OMERO (v5.6), using identical processing parameters for all image sets.

Two identical plates containing A549 cells treated with compounds 1, 4, or 5 (40 μM) were prepared for confocal microscopy experiments. One plate was incubated for 8 h, irradiated under normoxic conditions (21% O₂) with 460 nm light (16 J cm⁻²), and incubated for an additional 17 h prior to imaging. The second plate was kept under hypoxic conditions (1% O₂) in the dark for 24 h before imaging to minimize photoactivation. After image acquisition, the hypoxic plate was irradiated with 460 nm light (16 J cm⁻²) to evaluate light-dependent activation.

**Figure S90**: Confocal microscopy pictures of A549 lung cancer cells incubated with or without metal complexes **1**, **4**, or **5** (40 μM). Panels A–D show the brightfield, E-H show the green channel emission of the iridium compounds (λ_ex/em_ = 405 nm, λ_em_ = 450–635 nm), and panels I–L the merged images (brightfield + green channel). 1^st^ row – control with no compound; 2^nd^ row: cells treated with [IrCl(ppy)(terpy)]PF_6_ (**1**); 3^rd^ row: cells treated with [Ir(cys)(ppy)(terpy)]PF_6_ (**4**); 4^th^ row: cells treated with [Ir(sec)(ppy)(terpy)]PF_6_ (**5**). a) Cells were incubated with the metal complexes at 40 μM for 8 h in the dark, followed by irradiation with 460 nm light in normoxic conditions with 16 J/cm^2^ light dose and further incubation for 17h, then imaged; b) Cells were incubated with the metal complexes at 40 μM for 24 h in the dark and under hypoxic conditions to avoid activation of the complexes, then imaged; c) after imaging, the 96-well plate was irradiated in normoxic conditions with 460 nm light 16 J/cm^2^ light dose and immediately imaged again; images show the second imaging round.

# References

1.	D. N. Chirdon, W. J. Transue, H. N. Kagalwala, A. Kaur, A. B. Maurer, T. Pintauer and S. Bernhard, *Inorg. Chem.*, 2014, **53**, 1487-1499.

2.	V. V. L. Müller, P. V. Simpson, K. Peng, U. Basu, D. Moreth, C. Nagel, S. Türck, L. Oehninger, I. Ott and U. Schatzschneider, *Inorg. Chem.*, 2023, **62**, 16203-16214.

3.	G. R. Fulmer, A. J. M. Miller, N. H. Sherden, H. E. Gottlieb, A. Nudelman, B. M. Stoltz, J. E. Bercaw and K. I. Goldberg, *Organometallics*, 2010, **29**, 2176-2179.

4.	R. K. Harris, E. D. Becker, S. M. Cabral De Menezes, R. Goodfellow and P. Granger, *Concepts in Magnetic Resonance*, 2002, **14**, 326-346.

5.	G. M. Sheldrick, *Acta Crystallographica Section A Foundations and Advances*, 2015, **71**, 3-8.

6.	G. M. Sheldrick, *Acta Crystallographica Section A Foundations of Crystallography*, 2008, **64**, 112-122.

7.	A. M. Brouwer, *Pure Appl. Chem.*, 2011, **83**, 2213-2228.

8.	R. Ossola, O. M. Jönsson, K. Moor and K. McNeill, *Chem. Rev.*, 2021, **121**, 4100-4146.

9.	S. B. Partanen, P. R. Erickson, D. E. Latch, K. J. Moor and K. McNeill, *Environmental Science & Technology*, 2020, **54**, 3316-3324.

10.	X.-Q. Zhou, A. Busemann, M. S. Meijer, M. A. Siegler and S. Bonnet, *Chem. Commun.*, 2019, **55**, 4695-4698.

11.	E. Oliveros, P. Suardi-Murasecco, T. Aminian-Saghafi, A. M. Braun and H.-J. Hansen, *Helvetica Chimica Acta*, 1991, **74**, 79-90.

12.	R. Schmidt, C. Tanielian, R. Dunsbach and C. Wolff, *Journal of Photochemistry and Photobiology A: Chemistry*, 1994, **79**, 11-17.

13.	A. A. Abdel-Shafi, P. D. Beer, R. J. Mortimer and F. Wilkinson, *J. Phys. Chem. A*, 2000, **104**, 192-202.

14.	S. L. Hopkins, B. Siewert, S. H. C. Askes, P. Veldhuizen, R. Zwier, M. Heger and S. Bonnet, *Photochem. Photobiol. Sci.*, 2016, **15**, 644-653.

15.	M. D. Hall, K. A. Telma, K.-E. Chang, T. D. Lee, J. P. Madigan, J. R. Lloyd, I. S. Goldlust, J. D. Hoeschele and M. M. Gottesman, *Cancer Research*, 2014, **74**, 3913-3922.
